# Structural mechanism for nucleotide-driven remodeling of the AAA-ATPase unfoldase in the activated human 26S proteasome

**DOI:** 10.1038/s41467-018-03785-w

**Published:** 2018-04-10

**Authors:** Yanan Zhu, Wei Li Wang, Daqi Yu, Qi Ouyang, Ying Lu, Youdong Mao

**Affiliations:** 1 Center for Quantitative Biology, Peking University, Beijing, 100871 China; 2 State Key Laboratory for Artificial Microstructures and Mesoscopic Physics, Institute of Condensed Matter and Material Physics, School of Physics, Peking University, Beijing, 100871 China; 3 Intel Parallel Computing Center for Structural Biology, Dana-Farber Cancer Institute, Boston, MA 02215 USA; 4 Department of Cancer Immunology and Virology, Dana-Farber Cancer Institute, Department of Microbiology and Immunobiology, Harvard Medical School, Boston, MA 02115 USA; 5 Department of Systems Biology, Harvard Medical School, Boston, MA 02115 USA

## Abstract

The proteasome is a sophisticated ATP-dependent molecular machine responsible for protein degradation in all known eukaryotic cells. It remains elusive how conformational changes of the AAA-ATPase unfoldase in the regulatory particle (RP) control the gating of the substrate–translocation channel leading to the proteolytic chamber of the core particle (CP). Here we report three alternative states of the ATP-γ-S-bound human proteasome, in which the CP gates are asymmetrically open, visualized by cryo-EM at near-atomic resolutions. At least four nucleotides are bound to the AAA-ATPase ring in these open-gate states. Variation in nucleotide binding gives rise to an axial movement of the pore loops narrowing the substrate-translation channel, which exhibit remarkable structural transitions between the spiral-staircase and saddle-shaped-circle topologies. Gate opening in the CP is thus regulated by nucleotide-driven conformational changes of the AAA-ATPase unfoldase. These findings demonstrate an elegant mechanism of allosteric coordination among sub-machines within the human proteasome holoenzyme.

## Abstract (web summary)

The 26S proteasome consists of a core particle that is capped at each side by a regulatory particle. Here the authors present cryo-EM structures of the activated human 26S proteasome holoenzyme in three alternative open-gate states, which provides mechanistic insights into gate opening and dynamic remodeling of the substrate–translocation pathway.

## Introduction

The ubiquitin–proteasome system (UPS) participates in numerous important biological processes, such as regulation of gene expression, cell division, innate and adaptive immunity, and the response to proteotoxic stress^1–4^. A set of ubiquitylation pathways conjugate ubiquitin moieties on target proteins, which are then recognized and degraded by the 2.5-MDa 26S proteasome holoenzyme. The proteasome is composed of a 28-subunit barrel-shaped core particle (CP) and two 19-subunit regulatory particles (RP)^5–8^ capped at both sides of the CP. The catalytic chamber within the CP contains three proteolytically active threonine residues. Substrate entry into this chamber for degradation is controlled by the axial channel in the center of a heptameric α-ring, also called the CP gate, positioned on each side of the CP. Specific interactions of the α-ring with the RP or other activators may result in the opening of the CP gate^9–14^. The RP consists of two subcomplexes known as the lid and the base^15^. Recognition of a ubiquitylated substrate is mediated principally by ubiquitin receptors Rpn10 and Rpn13, the base subunits within the holoenzyme^4^. The globular domains of a substrate are mechanically unfolded by a heterohexameric ring-like subcomplex in the base consisting of six distinct subunits, Rpt1−6, which belong to the ATPases-associated-with-diverse-cellular-activities (AAA) family. To allow efficient substrate translocation, the conjugated ubiquitins are removed by the metalloprotease Rpn11, which is found in the lid.

Although major advances have been made in the last three decades in our understanding of the proteasome architecture^2,3,5–9,16–27^, only recently was the structure of the complete, intact proteasome in a resting state resolved at near-atomic resolution, so that a reliable Cα-backbone could be traced with partial assignment of amino acids^13,14,28–30^. Two other conformational states of the yeast proteasome were also resolved at similar resolutions lately^14,30^. However, all these high-resolution structures show the CP gate in a closed conformation, which must be opened to allow substrate degradation. We recently discovered that half of the complete human proteasome assembly, which was referred to as the RP–CP subcomplex including half of the CP in complex with the RP, can assume four major conformations under common adenosine triphosphate (ATP)-saturated solution condition, designated as the ground (S_A_), the commitment (S_B_), the gate-priming (S_C_), and the open-gate (S_D_) states^13^. Only the S_A_ state was resolved at a high resolution, whereas the resolutions of the other states remain relatively low. Except for the S_D_ state, all other states were found to comprise closed CP gates. In addition to earlier cryo-electron microscopy (cryo-EM) studies revealing that the yeast proteasome may assume three conformational states (s1, s2, and s3)^5–8,27,31–33^, the existence of the fourth state s4, analogous to the human S_D_ state, with similar structural features such as an open gate in the CP, was recently shown in the yeast proteasome at 7.7-Å resolution^14^. However, the insufficient resolution of the S_D_ and s4 states significantly limits our understanding of proteasome activation as well as the structural mechanism of substrate degradation^13,14^.

Here, we report near-atomic resolution cryo-EM structures of the activated human proteasome in complex with adenosine 5′-[γ-thio]triphosphate (ATP-γ-S), in which the RP–CP subcomplex samples three alternative conformational states (designated S_D1_, S_D2_, and S_D3_) when the CP gate is open. The three structures mainly differ in their AAA-ATPase ring, in which different nucleotide-binding patterns were observed to associate with conformational changes of the base. This allows us to visualize remarkable architectural transitions of the pore loops along the substrate–translocation pathway between the spiral-staircase and saddle-shaped-circle topologies. Taken together, these findings demonstrate an elegant mechanism of allosteric coordination among the sub-machines within the proteasome holoenzyme that actively binds nucleotides in preparation for substrate processing.

## Results

### Cryo-EM structure determination of the open-gate proteasome

**Fig. 1:**
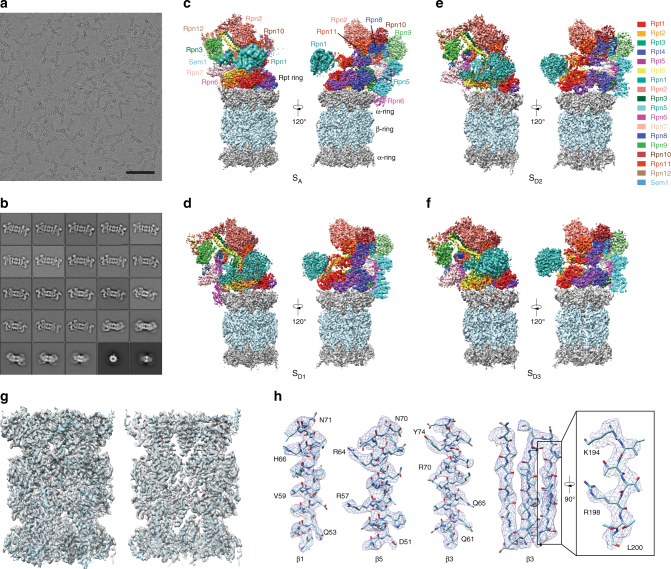
Cryo-EM structure determination of the ATP-γ-S-bound human proteasome. **a** A typical cryo-EM micrograph of the ATP-γ-S-bound human proteasome imaged with a Tecnai Arctica microscope equipped with a Gatan K2 Summit direct detector camera. Scale bar, 100 nm. **b** Typical reference-free 2D class averages computed using the ROME software^35^. **c**–**f** Two different views of the cryo-EM density maps in the S_A_ (panel **c**), S_D1_ (panel **d**), S_D2_ (panel **e**), and S_D3_ (panel **f**) states, colored according to subunit. **g** The 3.5-Å cryo-EM density of the CP in the combined S_D_ state superimposed over its atomic model. Left, a lateral perspective. Right, a central slice. **h** Typical high-resolution densities of the secondary structures in the cryo-EM structure of the CP in the open-gate S_D_ state

We collected cryo-EM data of the human proteasome holoenzyme in the presence of 1 mM ATP-γ-S, using a Gatan K2 Summit direct electron detector mounted on a 200-kV cryogenic electron microscope Tecnai Arctica (Fig. 1a and Supplementary Fig. 1). Using a deep-learning-based method^34^, we automatically extracted 502384 single-particle images from 8463 drift-corrected micrographs. After unsupervised 2D classification (Fig. 1b), 419,237 single-particle images were verified and chosen for further analysis^35^. The single-particle images of doubly capped proteasomes were then converted into single-particle images of RP–CP subcomplex by a 3D soft mask excluding one of the two RPs in image alignment and classification. Ultimately, we used exhaustive unsupervised focused 3D classification^36^ to obtain six conformations that were separately refined to their best resolutions (Supplementary Fig. 2 and Supplementary Table 1). Three of these correspond to the previously reported S_A_, S_B_, and S_C_ states of RP–CP subcomplex, which were refined to nominal resolutions of 3.6, 7.0, and 5.8 Å, respectively (Fig. 1c, Supplementary Figs. 1 and 3). The other three conformations, S_D1_, S_D2_, and S_D3_, all featuring an open CP gate, were refined to nominal resolutions of 4.2, 4.3, and 4.9 Å, respectively (Fig. 1d–f, Supplementary Figs. 1 and 3). To improve the resolution of the CP structure in the open-gate conformation, we combined the datasets of S_D1_, S_D2_, S_D3_, and another S_D_-like open-gate class and performed a high-resolution refinement focusing on the CP component, by applying a local mask that retains the CP structure (Supplementary Fig. 2). This allowed us to improve the open-gate CP structure in the S_D_-like states to an average resolution of 3.5 Å (Fig. 1g, h, Supplementary Figs. 1 and 3). Based on the atomic model of the S_A_ state, we built and refined three atomic models for S_D1_, S_D2_, and S_D3_, respectively (Supplementary Figs. 3 and 4). Consistent with published S_A_ structures, the ubiquitin receptor Rpn13 and the ubiquitin-interacting motif (UIM) of the ubiquitin receptor Rpn10 were missing in the cryo-EM densities in all conformational states^19,21^.

### Conformational states of the ATP-γ-S-bound human proteasomes

The structure of the ATP-γ-S-bound RP–CP subcomplex in the S_A_ or S_C_ state was nearly identical to the ATP-bound conformation in the same state^13^ (Supplementary Fig. 5a, c). The S_B_ state also closely resembles the previously reported S_B_ state, but shows a rather small rotation of RP against the interface between Rpn2 and the OB ring (Supplementary Fig. 5b). Consistent with our previous report, the S_A_, S_B_, and S_C_ states of the ATP-γ-S-bound RP–CP subcomplex all showed a closed CP gate^13^.

The S_D1_, S_D2_, and S_D3_ states all exhibit defined differences in the AAA-ATPase module as compared to the previously reported S_D_ state^13^ (Supplementary Fig. 6a–c). When the three datasets were combined to produce a merged cryo-EM reconstruction, the resulting map closely reproduced the previously reported S_D_ conformation^13^ (Supplementary Fig. 6d, f). In contrast to the improved resolution of the CP in the combined S_D_ map, the resolution of the AAA-ATPase module was limited to 5–6 Å in the combined S_D_ map, and was lower than those achieved in the separately refined S_D1_, S_D2_, and S_D3_ maps. Moreover, the AAA-ATPase module in the combined reconstruction also exhibited reduced densities than the lid subcomplex, indicating greater structural heterogeneity in the AAA-ATPase than in the other components. Separation of the three datasets improved the AAA-ATPase resolution, albeit at the expense of significantly reducing the particle numbers. These observations indicate that the previously reported S_D_ state is heterogeneous, and is most likely an average of multiple conformations^13^.

The probability of observing an open gate in the CP was dramatically enhanced in the presence of ATP-γ-S. The S_A_, S_B_, S_C,_ and S_D_ states of the ATP-bound human proteasome represented 76.1, 10.2, 5.8, and 7.9% of the entire particle population, respectively^13^. By contrast, the S_A_, S_B_, S_C_, S_D1_, S_D2_, and S_D3_ states represented 51.8, 3.5, 5.3, 14.9, 17.0, and 7.5% of the particle populations in the presence of ATP-γ-S, respectively. Thus, the probability for a human proteasome to adopt an S_D_-like state in the presence of ATP-γ-S is about five times higher than in the presence of ATP. Binding of the slowly hydrolyzable nucleotide analog ATP-γ-S instead of ATP apparently changed the conformational equilibrium among the coexisting states in favor of the S_D_-like states.

### Asymmetrical gate opening in the CP

**Fig. 2:**
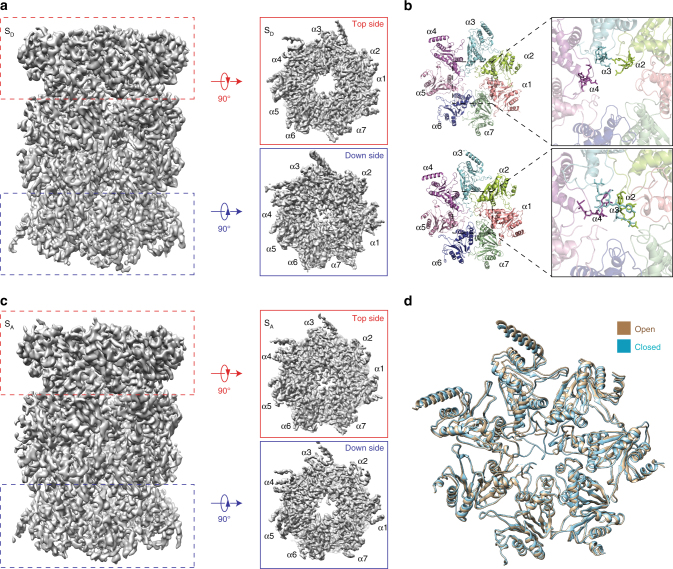
Asymmetric opening of the CP gates corresponding to the S_A_ and S_D_ states of the RP–CP subcomplex. **a** The side view (left), top view (upper right), and bottom view (lower right) of the CP density map in the combined S_D_ state. The upper half of the CP is associated with the RP in the S_D_ state of the RP–CP subcomplex. **b** The atomic models of two different α-rings from the perspective of the AAA-ATPase or the RP–CP interface in S_D_ in a cartoon representation (left). Close-up view of the central parts of the α-rings, showing that the CP gate is open on the top (upper right) and closed on the bottom (lower right). **c** The side view (left), top view (upper right), and bottom view (lower right) of the CP density map in the S_A_ state. The upper half of the CP is associated with the RP in the S_A_ state of the RP–CP subcomplex. **d** Superimposed atomic models of two different α-rings in a cartoon representation in the S_D_ state colored light orange (closed) and light blue (open)

The CP structure in the resting state of the ATP-bound human proteasome exhibited a *C2* symmetry^13,28,29^. However, this symmetry was broken in the ATP-γ-S-bound human proteasome. In the CP of the three S_D_ states, we observed asymmetrical gate opening (Fig. 2a). While the α-ring in contact with the RP in the S_D_ states was open in the center, the other α-ring in the opposite side of CP remained mostly closed at the same level of the density contour (Fig. 2a). Interestingly, in the S_A_ map, where the α-ring in contact with the RP was closed, the opposite side of the CP was instead mostly open (Fig. 2c). Thus, in many of the individual ATP-γ-S-bound proteasome holoenzyme structures, the CP gate opening did not take place in both α-rings simultaneously. By classifying the doubly capped holoenzymes, we found that ~46% of the particle population had only one of the two CP gates open, whereas ~39 and ~15% of the particle population had their CP gates closed and open on both sides, respectively (Supplementary Figs. 2 and 7).

The CP gate is principally controlled by the N-terminal tails of the α2 and α4 subunits; and the N-terminal tail of the α3-subunit behaves as a lynchpin of the gate, stabilizing the closed state of the CP gate^37^. Reconfiguration of the α3 tail is controlled by Rpt2 (ref. ^38^). The reorientation of these tails constitutes gating and controls substrate entry into the CP. Previous studies suggested that gate opening in the archaeal CP coincides with a prominent rotation in the α-subunits^39^. However, although the N-terminal tails of α2, α3, and α4 were rotated over a large angle to roughly align along the heptameric axis to open the CP gate (Fig. 2b), the helical elements connected to the gate-blocking tails in the α-subunits were nearly identical to those in the closed CP (Fig. 2d). This observation was consistent with the crystal structures of the yeast 20S proteasome in complex with the 11S regulators^40,41^ as well as the s4 state of the yeast 26S proteasome^14^.

### Conformational variation of the RP in the open-gate states

**Fig. 3:**
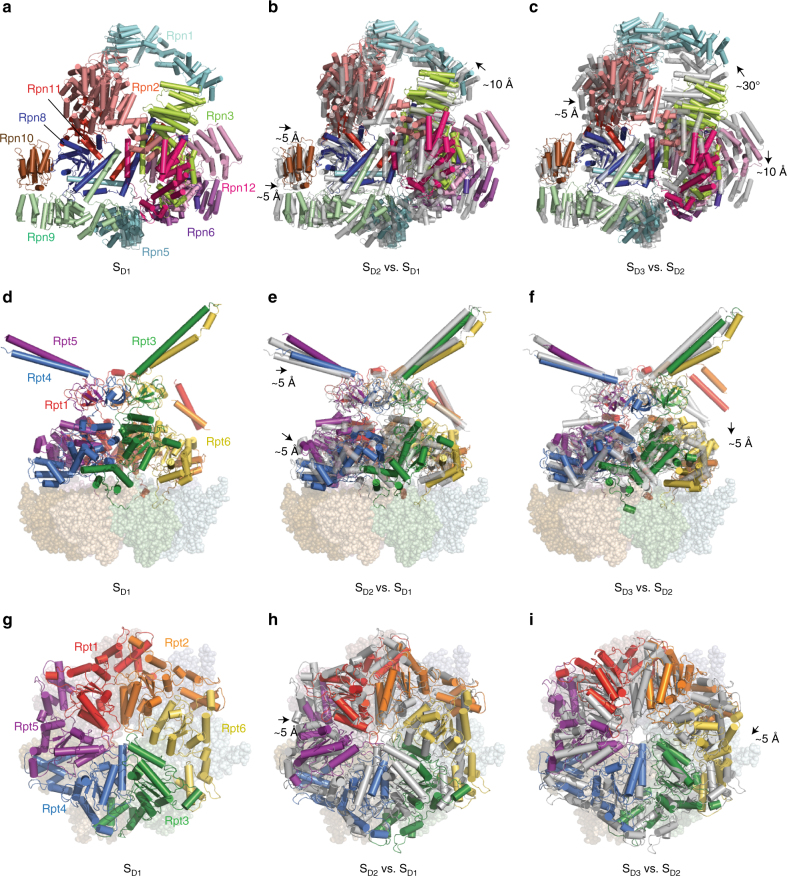
Conformational changes of the ATP-γ-S-bound RP in three different S_D_-like states. **a** Top view of the lid in S_D1_. **b**, **c** Top view of the lid in S_D2_ (**b**) and S_D3_ (**c**) superimposed over the transparent cartoons of S_D1_ and S_D2_, respectively. **d** Side view of the ATPase ring above the α-ring in S_D1_. **e**, **f** Side view of the ATPase ring above the α-ring in S_D2_ (**e**) and S_D3_ (**f**) superimposed with the transparent gray cartoons of S_D1_ and S_D2_, respectively. **g** Top view of the ATPase ring in S_D1_. **h**, **i** Top view of the ATPase ring in S_D2_ (**h**) and S_D3_ (**i**) superimposed over the gray cartoons of S_D1_ and S_D2_, respectively

The RP in the three open-gate states rotated and translated above the CP, with its subunits undergoing differential movements (Fig. 3, Supplementary Fig. 8 and Supplementary Movie 1). Relative to the S_D1_ state, the lid subcomplex of S_D2_ was translated overall for 1–2 nm toward Rpn3 and Rpn12 (Fig. 3a, b). The hexameric AAA-ATPase ring traveled ~1 nm above the heptameric α-ring in the direction along the lid translation (Fig. 3d–i), whereas Rpn1 was translated in the opposite direction for about 1 nm. The lid subcomplex further rotated ~5° clockwise in S_D3_ relative to S_D2_ (Fig. 3b, c). By contrast, Rpn1 rotated ~30° counterclockwise in S_D3_ relative to S_D2_. The AAA-ATPase ring overall moved ~1 nm backward in the S_D3_-to-S_D2_ transition as opposed to the S_D2_-to-S_D1_ transition.

The lateral RP–CP interfaces, which have an ancillary function in the regulation of the conformations of the axial substrate–translocation pathway within the holoenzyme^13^, also demonstrated prominent fluctuations. In the S_D1_ state, the amino (N)-terminal proteasome-cyclosome-initiation factor (PCI) domain of the lid subunit Rpn6 directly contacted the CP subunit α2 (Supplementary Fig. 6g). However, it was displaced ~20 Å away from this interface in the S_D2_ state, leaving the Rpn6 PCI domain mostly dissociated from the α2-subunit (Supplementary Fig. 6h). This interface seems to be partly re-established in S_D3_ (Supplementary Fig. 6i). However, the N-terminal PCI domain of Rpn6 displayed a marked reduction in density compared to the one in S_D1_, suggesting instability of the interface in S_D3_.

Rpn1 spans a large interface with the Rpt1–Rpt2 coiled-coil domain, with additional contacts with the AAA domain of Rpt2 stabilizing its association with the ATPase module^13,28,29^ (Supplementary Fig. 9a–c). It rotates about 45° clockwise around the AAA hexameric axis in both S_D1_ and S_D2_ relative to S_A_ (Supplementary Fig. 9d, e). Superposition of the Rpn1–Rpt2–Rpt1 structures in S_D1_ and S_D2_ indicates that the largest movement of Rpn1 happens in its N-terminal domain (Supplementary Fig. 9a–c). The movement of Rpn1 in the direction opposite to the motion of the AAA ring is conferred by the inter-domain rearrangement of the Rpt2 subunit, which is dependent on its nucleotide-binding state (see below).

### The RP–CP interface stabilizes the open gate of the CP

**Fig. 4:**
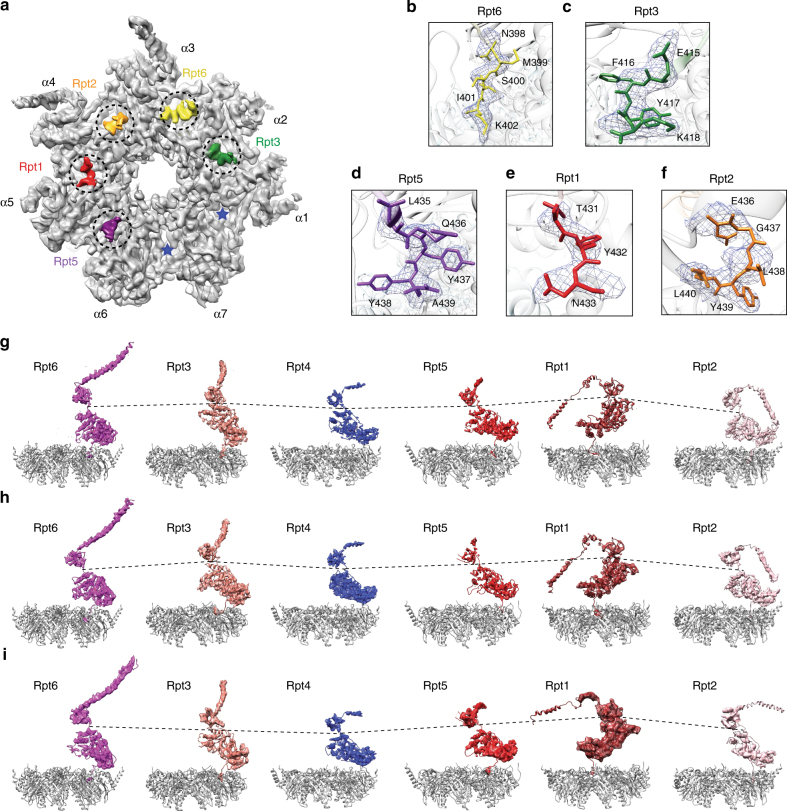
RP–CP interactions regulate the gate opening. **a** Overview of the RP–CP interface in which the C-terminal tails of Rpt1, Rpt2, Rpt6, Rpt3, and Rpt5 are shown to insert into the corresponding α-pockets in the S_D_ states. Two empty α-pockets are marked by asterisks. **b**–**f** Close-up views of each C-terminal tail shown in a stick representation superimposed over their corresponding cryo-EM densities shown in a mesh representation. **g**–**i** The Rpt subunits in cartoon representation superimposed with cryo-EM densities showing the vertical positions of each Rpt subunit relative to the α-ring in a gray cartoon representation in the S_D1_ (**g**), S_D2_ (**h**), and S_D3_ (**i**) states

In the previously published 8-Å map of the S_D_ state, we observed that the carboxyl (C)-terminal tails of Rpt subunits, with the exception of Rpt4, were inserted into the corresponding α-pockets^13^. However, the resolution was too low to provide a reliable atomic modeling of the Rpt C-terminus in the previously reported S_D_ map. The corresponding state of the yeast proteasome, s4, had a similar resolution, leaving the perceived difference between the S_D_ and s4 states unexplained regarding the RP–CP interface^14^. In the current study, the 3.5-Å map of the CP in the combined S_D_ state was substantially improved and thus allowed near-atomic model fitting of the C-terminal tails of the Rpt subunits (Fig. 4a–f, Supplementary Fig. 10). These C-terminal densities were also consistently observed in the cryo-EM maps of the S_D1_, S_D2_, and S_D3_ states at slightly lower resolutions. This observation suggests that the interactions between the C-termini of the Rpt subunits and the α-pockets are invariant among S_D1_, S_D2_, and S_D3_. Our high-resolution structural data thus substantiate the hypothesis that the five C-terminal tails of Rpt1, Rpt2, Rpt3, Rpt5, and Rpt6 are inserted into the α-pockets to stabilize the open gate of the CP.

### Structural rearrangement in the AAA-ATPase unfoldase

The ATPase ring in the resting state adopts a spiral “split lockwasher” architecture, in which the spiral ring is split at Rpt6, with other Rpt subunits organized from the highest to the lowest position relative to the CP in the sequence of Rpt3–Rpt4–Rpt5–Rpt1–Rpt2 (refs. ^13,28,29,42^). In the open-gate states, this spiral order with a single-split at Rpt6 was disarranged (Fig. 4g–i). Specifically, Rpt1 rose to the height of Rpt3, while Rpt4 dropped to the lower level around the position of Rpt2. Rpt6 also dropped with Rpt2 in S_D1_ and S_D2_, but rose to the higher level around the position of Rpt3 in S_D3_. Rpt2 demonstrated a prominent inter-domain motion between its small and large AAA subdomains, and fell back to the lowest position in S_D3_. Either Rpt2 or Rp4 subunits is situated at the lowest position, whereas Rpt1 or Rpt3 is located at the highest position. This rearrangement results in a novel ATPase ring architecture that follows a circle-on-a-saddle topology.

### Nucleotide states in the open-gate conformations

The nucleotide-binding pockets of the AAA family proteins share a common architecture and are surrounded by the highly conserved Walker A, Walker B, sensor I, sensor II, and arginine finger motifs^43^. The nucleotide binds the Walker A motif next to a short linker between the small and large AAA subdomains of the ATPases. The changes of the nucleotide’s chemical state, between ATP, ADP-Phosphate, and ADP, modify the geometric relationship between the small and large AAA subdomain, thus regulating the conformations of the Rpt subunits^32,44,45^. In both human and yeast proteasomes in the resting state, all six nucleotide-binding sites are occupied^13,14,28–30^. Consistent with these observations, we identified nucleotide densities in all six Rpt subunits in the density map of the S_A_ state of our ATP-γ-S-bound proteasome (Supplementary Fig. 11a, b).

**Fig. 5:**
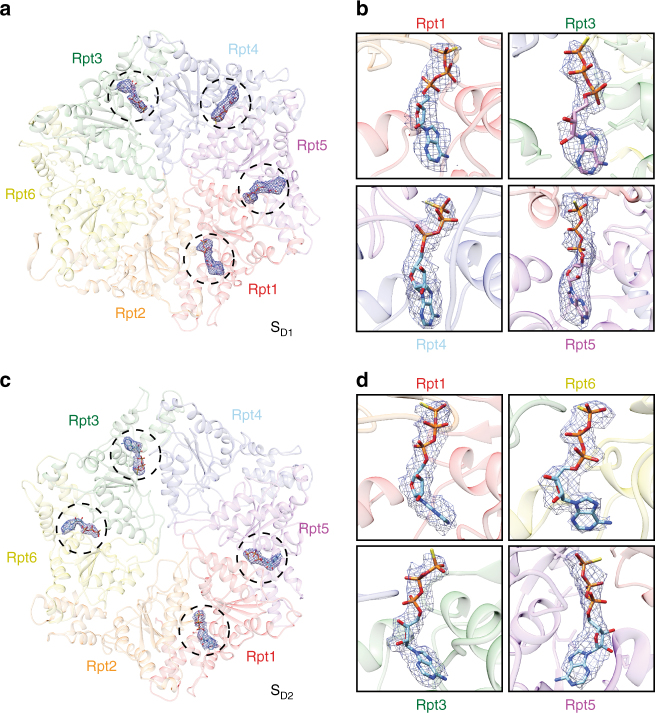
Cryo-EM densities of the nucleotides in the S_D1_ and S_D2_ states of the ATP-γ-S-bound RP–CP subcomplex. **a**, **c** Overview of the nucleotide-binding sites in the AAA-ATPase heterohexamer of the ATP-γ-S-bound human 26S proteasome in the S_D1_ (**a**) and S_D2_ (**c**) states. Bound nucleotides are shown in a stick representation superimposed over their respective cryo-EM densities in a blue mesh representation. **b**, **d** Close-up views of nucleotide conformations in the nucleotide-binding sites in the S_D1_ (**b**) and S_D2_ (**d**) states. ATP-γ-S was modeled into the nucleotide densities of four Rpt subunits in each state

In contrast to the full occupancy of the nucleotide-binding sites in the S_A_ state, we found partial occupancy in the open-gate states. In the S_D1_ state, we only observed ordered nucleotide densities in Rpt1, Rpt3, Rpt4, and Rpt5 (Supplementary Fig. 11c). The potential nucleotide densities in Rpt2 and Rpt6 appear to be very weak, suggesting either a partial occupancy or disordered nucleotide configuration within these nucleotide-binding sites (Fig. 5a and Supplementary Fig. 11c). Similarly, in the S_D2_ state, we observed well-ordered nucleotide densities in Rpt1, Rpt3, Rpt5, and Rpt6, but not in Rpt2 and Rpt4 (Fig. 5b and Supplementary Fig. 11d). Taken together, these data support the notion that nucleotide release and exchange might well take place in Rpt2, Rpt4, and Rpt6 during conformational transitions among these open-gate states, as well as between the S_D_-like states and the closed-gate states such as S_A_.

The release of a nucleotide in an ATPase protein destabilizes the nucleotide-bound conformation, inducing large structural arrangements between the small and large AAA subdomains^46^. In line with the observations of disordered nucleotide densities, the Rpt2 and Rpt6 densities in the S_D1_ map, the Rpt2 density in the S_D2_ map, and the Rpt6 density in the S_D3_ map were of lower quality at slightly lower local resolutions than the other Rpt subunits in the same map, indicating that their conformations are destabilized, potentially due to nucleotide release (Supplementary Fig. 11c, d).

### Remodeling of the substrate–translocation pathway

**Fig. 6:**
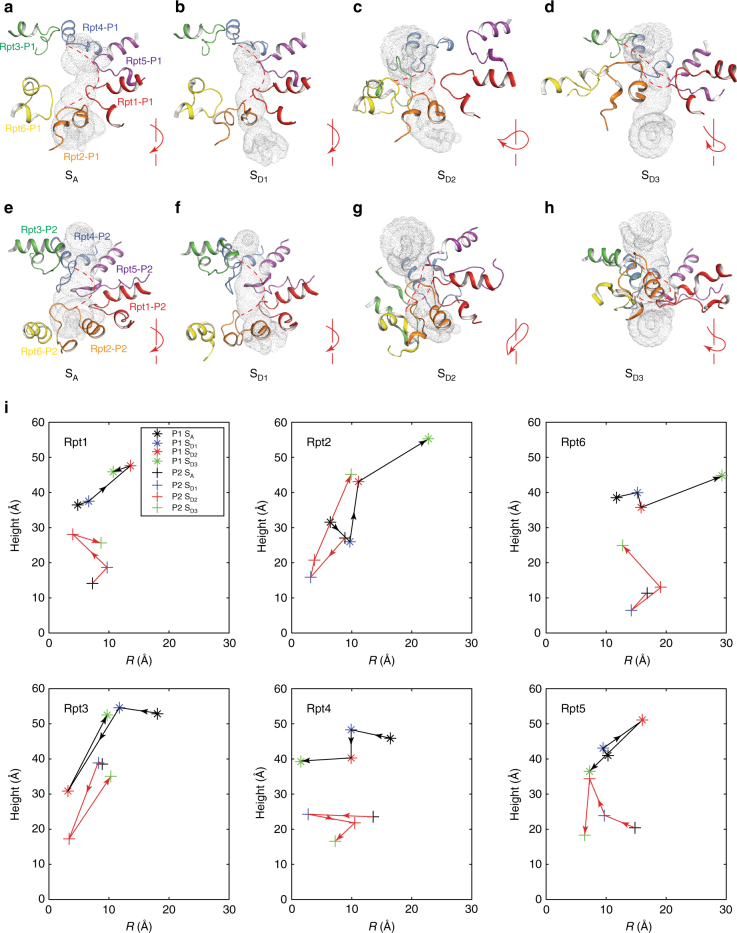
Topological remodeling of the pore loops along the substrate–translocation channel. **a** Close-up view of the pore-1 loops from six Rpt subunits constricting the AAA channel in the S_A_ state. The solvent accessible surface of the AAA channel was estimated using the program HOLE and is shown in gray dots. The AAA channel is aligned top-down vertically, whereby the substrates are supposed to enter from the top end of the pathway. **b**–**d** Close-up views of the pore-1 loops from six Rpt subunits constricting the AAA channel in the S_D1_, S_D2_, and S_D3_ states. The lower right inset in each panel shows an illustrative graph summarizing the pore-1 loop organization topology. The arrows show the clockwise direction of oligomeric ATPase organization from Rpt3, through Rpt4, Rpt5, Rpt1, and Rpt2, to Rpt6. The pore-1 loops in S_D2_ and S_D3_ show saddle-like topologies versus the spiral topology in S_D1_ and S_A_. The AAA channels are aligned to that shown in **a** based on their positions relative to the common reference of the CP. **e** Close-up view of the pore-2 loops from six Rpt subunits decorating the AAA channel in the S_A_ state. **f-h**, **g** Close-up view of the pore-2 loops from six Rpt subunits constricting the AAA channel in the S_D1_, S_D2_, and S_D3_ states. **i** The relative positional changes of each pore loop in the S_A_, S_D1_, S_D2_, and S_D3_ states. The arrows show the direction of the movements in the pore loops during state transitions from S_A_ to S_D1_(**f**), S_D2_(**g**), and S_D3_(**h**), respectively. The vertical axis indicates the relative height of the pore loops along the axis of the ATPase channel and the CP gate. The horizontal axis indicates the lateral distance orthogonal to the channel axis

The central channel formed by the hexameric ring comprising the AAA domain of the Rpt subunits is narrowed by inward-facing pore loops that were thought to drive the translocation of substrates^25,46,47^. In the S_A_ state, the pore-1 and pore-2 loops from neighboring subunits were found to pair with each other, constituting the constrictions of the AAA channel^13^. The hydrophobic residues in the pore-1 loops of Rpt4, Rpt5, and Rpt1 are paired with charged residues in the pore-2 loops of Rpt3, Rpt4, and Rpt5, respectively^13^. Although the AAA-ATPase module in the S_D1_ state was in a conformation with its nucleotide-binding configuration quite different from that in S_A_, the pore-loop pairing architecture in S_D1_ resembles the one in S_A_. By contrast, in the S_D2_ and S_D3_ states, the pore loops were dramatically reorganized into tilted, saddle-shaped circles (Fig. 6 and Supplementary Fig. 12). The saddle-like circle formed by the pore loops in S_D2_ was tilted toward the direction opposite to that in S_D3_, which is compatible with the overall wobbling motion of the ATPase ring.

Driven by the nucleotide release in Rpt2 and Rpt3, the pore-1 and pore-2 loops of Rpt2 and Rpt3 are moved down in S_D2_ and up in S_D3_, traveling over the longest distance of about 40–60 Å from S_D1_ to S_D3_ (Fig. 6i, Supplementary Movies 2 and 3). By contrast, both pore-1 and pore-2 loops in Rpt1 and Rpt4 travel down over much shorter distances (~20–30 Å) in the state transition from S_D1_ to S_D2_ and to S_D3_. Additional pore loops, most prominently in Rpt2, Rpt3, and Rpt5, alternate up and down in the triple-state transition. In contrast to the vertical movements in most pore loops, the pore loops in Rpt4 mostly move laterally with distances of around 10 Å.

In the S_A_ state, the pore-loop paring along the axial channel of the AAA ring makes a passage that is too narrow to smoothly translocate even an unfolded polypeptide^13,28,29^. The constrictions narrowed by the pore loops are substantially reconfigured in the S_D2_ and S_D3_ structures, so that the AAA channel is widened overall relative to that in the S_D1_ state. However, the narrowest constrictions in S_D1_, S_D2_, and S_D3_ are still too narrow to allow the translocation of peptides with large aromatic side chains such as tryptophan (Supplementary Fig. 13), suggesting that conformational flexibility in the pore loops might be necessary to accommodate substrate translocation^46^. Indeed, although the pore loops of Rpt6 do not contribute to the constriction of the AAA channel in S_D1_, they move in to directly shape the AAA channel in S_D2_. By contrast, the pore-1 loops of Rpt5 and Rpt6 are moved away from the substrate–translocation pathway in S_D2_ and S_D3_, respectively (Fig. 6c, d), whereas the pore-2 loops of Rpt5 and Rpt6 remain engaged in the formation of the AAA channel constriction in S_D2_ and S_D3_ (Fig. 6g, h). Hence, the architectural remodeling of the substrate–translocation channel observed in the three S_D_-like states manifests a marked conformational plasticity of the pore loops which is presumably necessary for substrate unfolding.

## Discussion

In this work, we solved the structures of three distinct open-gate states of the activated human proteasome at high resolution, expanding the number of known coexisting conformational states from four to six. Consistent with a previous study on the ATP-γ-S-bound yeast proteasome^32^, the conformational states of the human proteasome were redistributed upon the binding of slowly hydrolyzable ATP-γ-S as opposed to ATP. All previous studies and the present work suggest that full occupancy of six nucleotide-binding sites stabilizes the resting state, in which the CP gate is closed in both human and yeast proteasome holoenzymes^13,14,28–30^. Full occupancy of ATP-γ-S in all nucleotide-binding sites may be less favorable, allowing more copies of the complex to assume the S_D_-like states. Nonetheless, the S_A_ state was still the dominant population (~50%) among the ATP-γ-S-bound human proteasome structure snapshots.

Importantly, we observed different patterns of nucleotide binding in the AAA-ATPase heterohexamer when the CP gate is open. We found that at least four nucleotide-binding sites are occupied in the S_D_-like open-gate states. This is in line with previous biochemical studies suggesting that four nucleotides are needed for the functioning of the PAN (proteasome-activating nucleotidase) AAA-ATPase in archaea^48^. Similar to our S_D1_ state, a recent study on the yeast proteasome in complex with ADP-aluminum fluoride (AlF_x_) showed that the apo-like states in Rpt2 and Rpt6 allow the ATPase to adopt a different conformation when the CP gate is still closed^30^. A pattern of nucleotide binding similar to our S_D1_ state was also observed in the yeast mitochondrial inner membrane AAA+ protease YME1 bound to a substrate polypeptide^49^. By contrast, the nucleotide-binding pattern of our S_D2_ state is similar to that of the caseinolytic protease X (ClpX) hexamer, another AAA+ unfoldase in *E. coli*, in which the two apo-like binding sites are in the subunits positioned opposite each other around the hexameric axis^46^. Notably, our observation of the apo-like state in Rpt2 and Rpt4 in the S_D2_ state is in line with previous mutagenesis studies, in which tyrosine-to-alanine mutations in pore-1 loops of Rpt2 and Rpt4 led to the biggest defects of substrate degradation^47^. To sum up, the compatibility of the nucleotide-binding patterns in the S_D_-like states of human proteasomes with a large body of literature^30,46–49^ prompts their relevance to the proteasome function.

The high-resolution structures of the open-gate states clarify the mechanism of CP gate opening in the human proteasome. The CP gate was previously observed to sample the open state with a low probability in the absence of the RP^50^. Thus, our structural data suggest that insertion of five Rpt C-tails into the α-pockets stabilizes the open-gate state of the CP. The open-gate s4 state of the yeast proteasome^14^, resembling our previously reported S_D_ state^13^, was recently visualized at relatively low resolution in the holoenzyme in complex with ADP-beryllium fluoride (BeF_*x*_) and ATP/BeF_*x*_. In contrast to the human S_D_ state, the C-terminal tails of Rpt1 and Rpt4 were not seen to dock into the α-pockets in the yeast s4 state^14^. This is different from our observation that only the Rpt4 C-tail is not inserted into the corresponding α-pocket in the open-gate human proteasome^13^. Indeed, our high-resolution cryo-EM maps of all three open-gate S_D_-like states consistently revealed the densities of five Rpt C-tails in the α-pockets, confirming the C-tail insertion of all Rpt subunits except Rpt4 into the α-ring of the CP. Our result is also reminiscent of a previously study on the chaperone-mediated assembly intermediate base–CP complex, in which the C-termini of Rpt6, Rpt2, and Rpt1 were seen to insert into α-pockets^51^.

The multiple conformations of the AAA-ATPase unfoldase associated with an open CP gate provide novel mechanistic insights into proteasome activation and how it prepares itself for substrate engagement. We speculate that substrate engagement must be coordinated with distributed conformational changes in the RP and the RP–CP interface. Binding of six nucleotides in the ATPase ring may lock the topological relationship between the RP and the CP in the S_A_ state, so that it does not permit a continuous movement of the AAA-ATPase module. To allow more Rpt C-terminal tails to reach their nearby α-pockets, which seems to be crucial for maintaining CP gate in the open state, the structural constraints preventing the continuous movement of the base must be relieved. Thus, the human proteasome undergoes dramatic conformational changes in the RP throughout the S_B_ and S_C_ states to attain the S_D_-like states^13^, in which the AAA-ATPase unfoldase gains certain freedom to simultaneously explore different conformations that are compatible with the initiation of substrate translocation. Given the similarity of pore-loop architecture between S_A_ and S_D1_, we speculate that the S_D1_ state likely represents the conformation in which the holoenzyme prepares to accept an incoming substrate via the pore loops, and that conformational change from S_D1_ to either S_D2_ or S_D3_ might be manifestation of the preferred movement of the pore loops to engage an incoming substrate. Thus, we envision that sustaining certain degree of AAA-ATPase dynamics in the absence of substrates might facilitate axial association of substrates with the AAA channel and a smooth transition into the substrate–translocation phase. We hypothesize that substrate binding with the pore loops might further expand the conformational landscape of the AAA-ATPase unfoldase, driving it to a greater level of conformational dynamics to meet the functional needs of converting the chemical energy of ATP hydrolysis into mechanical work in the form of axial, unidirectional movement of the engaged substrate. A comprehensive understanding of the functional cycle of the holoenzyme will require an in-depth analysis of the complete conformational spectrum and sequence of states of the substrate-engaged proteasome at high resolution.

## Methods

### Protein expression and purification

Human proteasomes were purified through affinity chromatography on a large scale from a stable HEK293 cell line expressing HTBH (hexahistidine, TEV cleavage site, biotin, and hexahistidine) tagged hRPN11 (a gift from L. Huang, Departments of Physiology and Biophysics and of Developmental and Cell Biology, University of California, Irvine, CA 92697)^52^. The cells were homogenized with a Dounce tissue grinder in a lysis buffer (50 mM NaH_2_PO_4_, pH 7.5, 100 mM NaCl, 10% glycerol, 5 mM MgCl_2_, 0.5% NP-40, 5 mM ATP and 1 mM DTT) containing protease inhibitor cocktail (Roche, Germany). The lysates were cleared, and incubated with the NeutrAvidin agarose beads (Thermo Fisher Scientific, MA, USA) overnight at 4 °C. The beads were washed by excess lysis buffer and then by the wash buffer (50 mM Tris-HCl pH 7.5, 1 mM MgCl_2_ and 1 mM ATP). Usp14 was removed from the proteasomes using the wash buffer containing 150 mM NaCl for 30 min. The proteasome holoenzymes were eluted from the beads through cleavage by TEV protease (Invitrogen, CA, USA). The doubly capped proteasome was further purified by gel filtration on a Superose 6 10/300 GL column (GE Healthcare, PA, USA) at a flow rate of 0.15 ml/min in the running buffer (30 mM Hepes pH 7.5, 60 mM NaCl, 1 mM MgCl_2_, 10% Glycerol, 0.5 mM DTT, 0.8 mM ATP). The gel-filtration fractions were concentrated to about 2 mg/ml. Immediately before cryo-EM sample preparation, the proteasome sample was buffer-exchanged into 50 mM Tris-HCl pH 7.5, 1 mM MgCl_2_, 1 mM ATP-γ-S, 0.5 mM TCEP, and 0.005% NP-40.

### Data collection

CryoEM sample grids were prepared using the FEI Vitrobot Mark IV (Thermo Fisher Scientific, MA, USA). C-flat grids (R1/1 and R1.2/1.3; 400 Mesh, Protochips, CA, USA) were glow-discharged before a 2.5-μl drop of 1.5 mg/ml proteasome solution was applied to the grids in an environment-controlled chamber with 100% humidity and temperature fixed at 4 °C. After 2 s of blotting, the grid was plunged into liquid ethane and then transferred into liquid nitrogen. The cryo-grids were imaged using an FEI Tecnai Arctica microscope (Thermo Fisher Scientific, MA, USA), equipped with an Autoloader and operating at an acceleration voltage of 200 kV at a nominal magnification of 235,000 times. Coma-free alignment was done manually to optimize the electron optics prior to data collection. Cryo-EM movie data were collected semi-automatically using Leginon^53^ software version 3.1 on a Gatan K2 Summit direct detector camera (Gatan Inc., CA, USA) in a super-resolution counting mode, with 7.5 s of total exposure time and 250 ms per frame. Each exposure resulted in a movie of 30 frames with an accumulated dose of 30 electrons/Å^2^. The calibrated physical pixel size and the super-resolution pixel size were 1.5 and 0.75 Å, respectively. The defocus was prescribed in the range from −0.7 to −3.0 μm. A total of 10,369 movies were collected, among which 8463 movies were selected for further data analysis after visual inspection of each image for quality.

### Cryo-EM data processing and reconstruction

All frames of the raw movies were first corrected for their gain using a gain reference recorded within 2 days of the acquired movie, after which they were shifted and summed to generate a single micrograph that was corrected for overall drift using the MotionCor2 program^54^. Each drift-corrected micrograph was used for the determination of the actual defocus of the micrograph using the CTFFind3 program^55^. Reference-free 2D classification was carried out in ROME 1.0 that combined the maximum-likelihood-based image alignment and statistical machine-learning-based classification^35^. 3D classification and high-resolution refinement were conducted in RELION 1.3 (ref. ^56^). In all, 502,384 complete particles of the proteasome were picked using DeepEM, a recently developed program based on a machine-learning algorithm^34^. The initial model was generated in EMAN2 (ref. ^57^). A subset of 15,044 particles was used for reference-free classification using e2refine2d.py and for the generation of an initial model using e2initialmodel.py.

All 2D and 3D classifications^35,56^ were done at a pixel size of 1.5 Å. After the first round of reference-free 2D classification with ROME^35^, bad particle classes were rejected upon inspection of class average quality, which left 419,237 particles. The initial model, low-pass filtered to 60 Å, was used as the input reference to conduct unsupervised 3D classification into four classes without assumption of any symmetry, using angular sampling of 7.5° and a regularization parameter *T* of 4 with RELION^56^. Three classes had both RP caps on while the other had a single RP cap. Two of the double-cap classes, which accounted for 45% of the particles, showed an open-gate feature while the third double-cap class and the single-cap class appeared to have both the CP gates in a closed state. The two groups were separated and each was further sorted by reference-free 2D classification^35^, and a total of 395,941 high-quality particles were selected from the two groups for the following analysis.

To reduce the irrelevant heterogeneity in our particle population due to conformational variations, and to improve the map resolution, each complete proteasome particle was split into two pseudo-single-cap particles by a mask including an RP and a complete CP^13,29^. We re-centered each particle to its new center calculated from the refined Euler angles and x/y-shifts. Accordingly, the center of each particle was shifted toward its RP part. We then shrank the box size and re-extracted the particles from raw micrographs. There were 377,313 pseudo-single-cap particles in the dataset of the open-gate states and 461,161 pseudo-single-cap particles in the dataset of the closed-gate state. Both datasets were further separately sorted by reference-free 2D classification, after which 370,068 and 421,814 high-quality particles were left in the closed- and open-gate datasets, respectively. The datasets of the open- and closed-gate states were then separately subjected to the second round of 3D classification, each into six classes. Five of the classes in the closed-gate states were similar in their overall conformations, and were combined with another two closed-gate classes classified from the “open-gate” dataset. The remaining single class from the closed-gate group had density that was partially averaged out, indicating large heterogeneity in the class. This class was further classified into four classes, three of which were abandoned, and the remaining high-quality class (29%) was added to the two classes (40%) of the open-gate states from the second round the 3D classification. The new open-gate datasets subsequently went through another two rounds of 3D classification and gave rise to three S_D_-like states and an S_C_ state with 66,246, 75,726, 33,278, and 23,567 particles, respectively. Another round of 3D classification of the closed-gate dataset into four classes gave rise to the S_A_ state containing 214,251 particles in three classes excluding those true singly-capped particles. A collection of the remaining 68,819 particles from both groups was subjected to another round of 3D classification into four classes and yielded the S_B_ state in the fourth class with 15,536 particles.

The final refinement of each state was done using the particle data in the counting mode with a pixel size of 1.5 Å. Based on the in-plane shift and Euler angle of each particle from the last iteration of refinement, we reconstructed the two half-maps of each state using single-particle images at the super-counting mode with a pixel size of 0.75 Å, which resulted in reconstructions of the S_A,_ S_B,_ S_C,_ S_D1,_ S_D2_, and S_D3_ states with overall resolutions of 3.6, 7.0, 5.8, 4.2, 4.3, and 4.9 Å, respectively, measured by gold-standard FSC at 0.143-cutoff on two separately refined half-maps. We then further combined the S_D1_, S_D2_, and S_D3_ datasets, and focused the refinement on the CP with a local mask, which yielded a 3.5-Å CP structure with one gate open and the other closed. To further improve the density map quality of the AAA-ATPase, we conducted focused refinement using a mask that kept the densities of the AAA-ATPase and the CP, which excluding the lid and non-ATPase subunits in the base, during the last several iterations of the refinement. This helped improve the gold-standard resolution of the AAA-ATPase and CP components in the S_D1_, S_D2_, and S_D3_ states to 4.0, 4.1, and 4.8 Å, respectively. Prior to visualization, all density maps were sharpened by applying a negative B-factor (Supplementary Table 1). Local resolution variations were further estimated using ResMap on the two half-maps refined independently^58^.

### Atomic model building and refinement

To build the initial atomic model of S_A_ and S_D1–3_ states, we used a previously published ATP-bound human 26S S_A_ structure and then manually improved the main-chain and side-chain fitting in Coot^59^ to generate the starting coordinate files. An exception was Rpn1 in the S_D1–3_ states, where homology modeling was conducted in Modeller^60^ using Rpn2 in the ATP-bound human 26S structure as a reference to generate a starting coordinate file. To fit the model to the reconstructed density map, we first conducted rigid-body fitting of the segments of the model in Chimera^61^, after which the fit was improved manually in Coot. Finally, each refinement of the atomic model was carried out in real space with program Phenix.real_space_refine^62^, with secondary structure and geometry restrains to prevent overfitting. Pseudo-atomic models of the S_B_ and S_C_ states were fitted in Coot starting from the ATP-bound human 26S S_B_ and S_C_ structures, respectively, and were further refined in real space using Phenix.real_space_refine with secondary structure and geometry restrains.

### Structural analysis and visualization

Structural comparison was conducted in Pymol^12^ and Chimera. The solvent accessible surface of the peptide-conducting channels in different states were separately calculated with respect to the OB ring and the AAA ring using HOLE^63^. All figures of the structures were plotted in Chimera^61^, Pymol^12^, or our Python code that employs the Matplotlib package.

### Data availability

The single-particle reconstructions and atomic coordinates reported in this paper have been deposited in the Electron Microscopy Data Bank, www.emdatabank.org (accession nos.: EMD-8662 for the CP structure in S_D_, EMD-8663, EMD-8664, EMD-8665, EMD-8666, EMD-8667, and EMD-8668 for the structures S_D1_, S_D2_, S_D3_, S_A_, S_B_, and S_C_, respectively) and worldwide Protein Data Bank, www.wwpdb.org (PDB ID codes: 5VFO for the CP structure of S_D_, 5VFP, 5VFQ, 5VFR, 5VFS, 5VFT, 5VFU for the ATP-γ-S-bound holoenzymes of the S_D1_, S_D2_, S_D3_, S_A_, S_B_, and S_C_ states, respectively). The raw micrographs and particle data have been deposited in the Electron Microscopy Pilot Image Archive, http://www.ebi.ac.uk/pdbe/emdb/empiar/ (accession no. EMPIAR-10090). Other data are available from the corresponding authors upon reasonable request.

## Electronic supplementary material

Supplementary Information

Peer Review Report

Description of Additional Supplementary Info

Supplementary Movie 1

Supplementary Movie 2

Supplementary Movie 3
