# Supplementary material for: Structural mechanism for nucleotide-driven remodeling of the AAA-ATPase unfoldase in the activated human 26S proteasome
Source: Nat Commun. 2018 Apr 10;9:1360. doi: 10.1038/s41467-018-03785-w (PMC5893597; doi:10.1038/s41467-018-03785-w)
Supplement: Supplementary file 1 — Supplementary Information [file 41467_2018_3785_MOESM1_ESM.pdf]

Supplementary Information

Structural mechanism for nucleotide-driven remodeling of the AAA-ATPase unfoldase in the activated human 26S proteasome

Zhu *et al*.

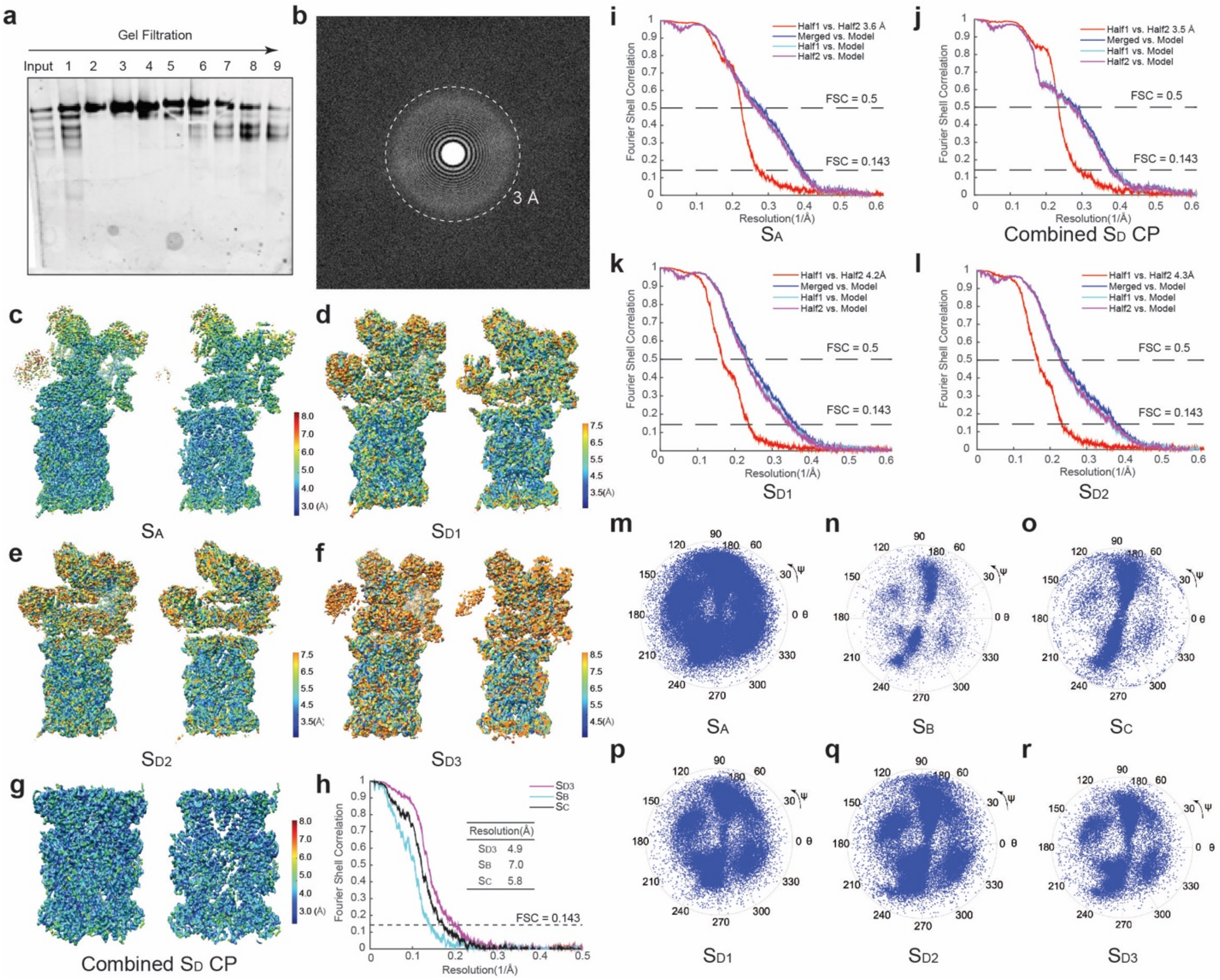

Supplementary Figure 1. Purification and cryo-EM structure determination of the ATP-γ-S-bound human 26S proteasome.

(**a**) Native gel electrophoresis analysis of fractions collected from a Superose 6 10/300 GL column that were rich in the ATP-γ-S-bound human 26S proteasome.

(**b**) The power spectrum of a typical drift-corrected micrograph at the super-resolution mode.

(**c**) Local resolution measurement of the S_A map by ResMap using two half-maps separately refined in a gold-standard procedure.

(**d**) Local resolution measurement of the S_{D1} map by ResMap using two half-maps separately refined in a gold-standard procedure.

(**e**) Local resolution measurement of the S_{D2} map by ResMap using two half-maps separately refined in a gold-standard procedure.

(**f**) Local resolution measurement of the S_{D3} map by ResMap using two half-maps separately refined in a gold-standard procedure.

(**g**) Local resolution measurement of the $S_{D1\text{-}3}$ combined 20S CP map by ResMap using two half-maps separately refined in a gold-standard procedure.

(**h**) The gold-standard FSC curves of the final refined cryo-EM maps in the S_{D3}, S_B and S_C state.

(**i**) FSC plots give an estimate of the resolution in the S_A state. Red curve shows the gold-standard FSC in the S_A state. Blue, cyan and purple curves show the FSC cross-validation using the atomic model against the two half-maps and the merged map in the S_A state.

(**j**) FSC plots give an estimate of the resolution of the CP density map refined from the dataset combining those of S_{D1}, S_{D2}, and S_{D3}. Red curve shows the gold-standard FSC for the CP density map in the combined S_D state. Blue, cyan and purple curves show the FSC cross-validation using the atomic model against the two half-maps and the merged map of the CP in the combined S_D state.
(**k**) FSC plots give an estimate of the resolution in the S_{D1} state. Red curve shows the gold-standard FSC in the S_{D1} state. Blue, cyan and purple curves show the FSC cross-validation using the atomic model against the two half-maps and the merged map in the S_{D1} state.
(**l**) FSC plots give an estimate of the resolution in the S_{D2} state. Red curve shows the gold-standard FSC in the S_{D2} state. Blue, cyan and purple curves show the FSC cross-validation using the atomic model against the two half-maps and the merged map in the S_{D2} state.
(**m**-**r**) Angular distributions of refined 3D reconstructions of the present S_A, S_B, S_C, S_{D1}, S_{D2} and S_{D3}, respectively.

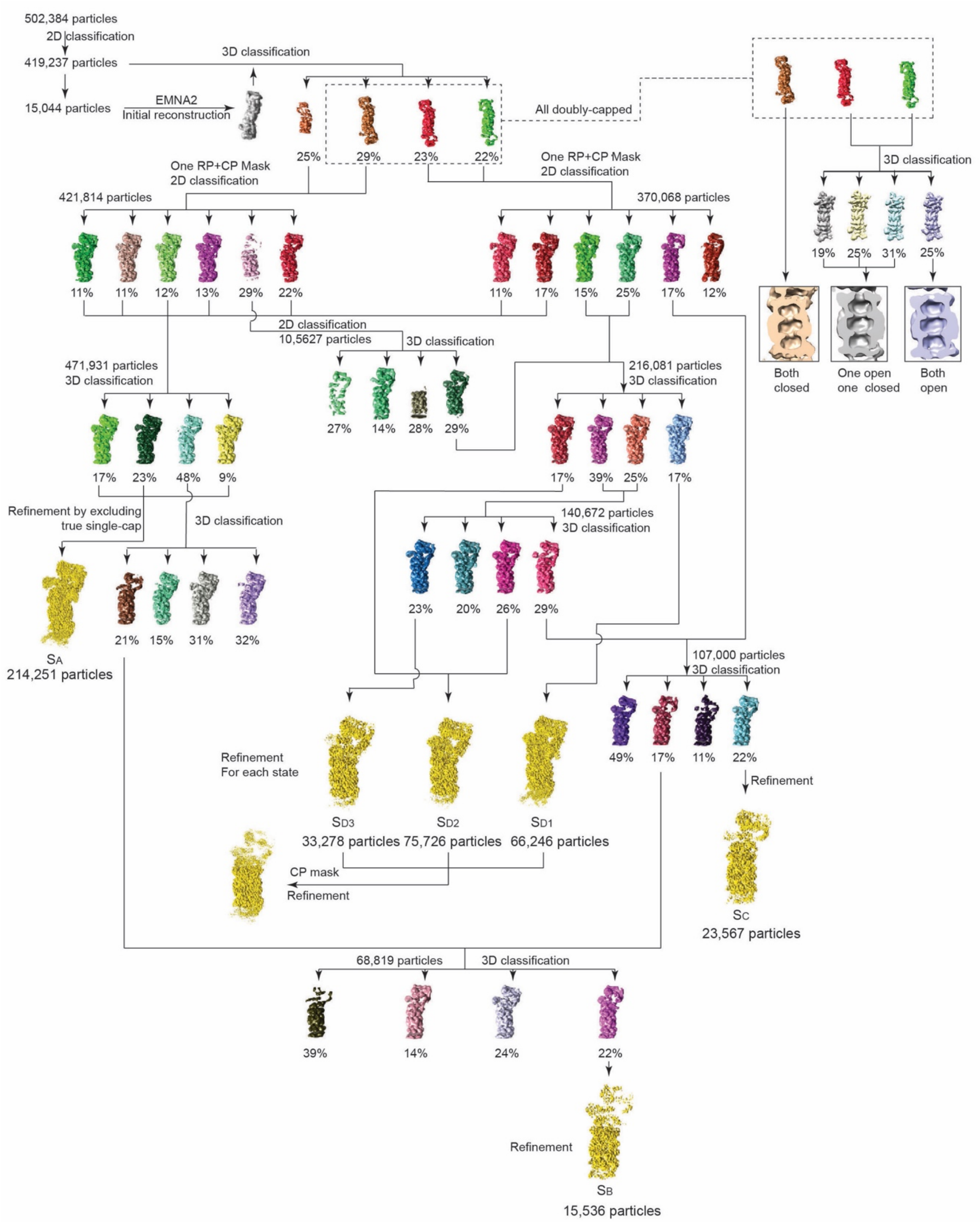

Supplementary Figure 2. 3D classification and refinement of a heterogeneous dataset into several homogeneous datasets for high-resolution reconstruction.

Schematic procedure of the maximum-likelihood based 2D and 3D classification that help to improve the homogeneity of the particle datasets of the pseudo-single cap. During the classification, the classes showing incomplete assemblies were discarded. The classes of complete assemblies with similar or same conformations were combined for either further classification or high-resolution refinement.

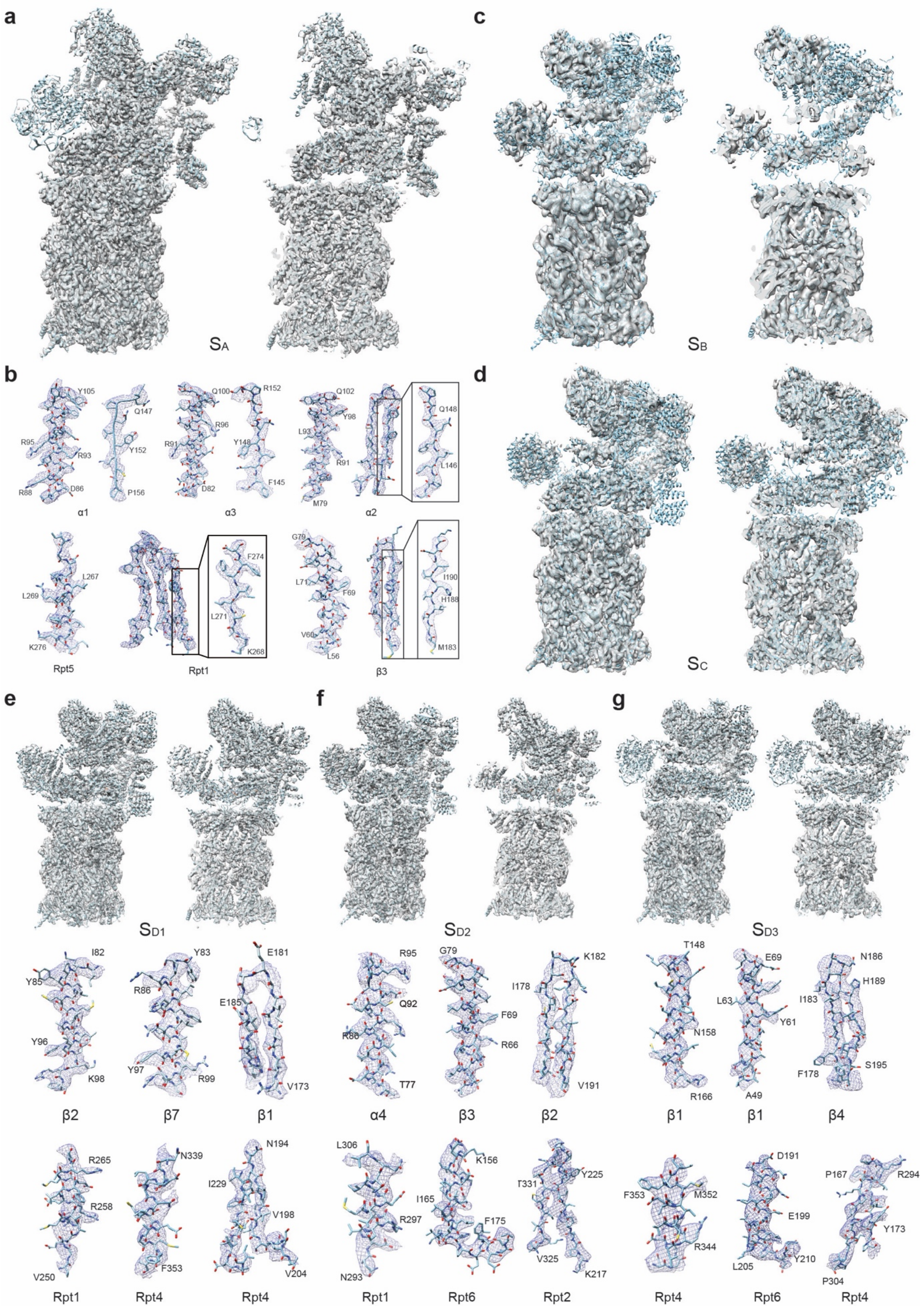
a
SA
b
Y105
Q147
R95
R93
Y152
R88
D86
P156
α1
Q100
R152
R96
R91
Y148
D82
F145
α3
Q102
Y98
L93
R91
M79
Q148
L146
α2
Rpt5
L267
L269
K276
Rpt1
F274
L271
K268
G79
L71
F69
V60
L56
I190
H188
M183
β3
c
SB
d
SC
e
SD1
I82
Y85
Y96
K98
β2
Y83
R86
Y97
R99
β7
E181
E185
V173
β1
R265
R258
V250
Rpt1
N339
F353
Rpt4
N194
I229
V198
V204
Rpt4
f
SD2
R95
Q92
R86
T77
α4
G79
F69
R66
β3
K182
I178
V191
β2
L306
R297
N293
Rpt1
K156
I165
F175
Rpt6
T331
Y225
V325
K217
Rpt2
g
SD3
T148
N158
R166
β1
E69
L63
Y61
A49
β1
N186
H189
I183
F178
S195
β4
F353
M352
R344
Rpt4
D191
E199
L205
Y210
Rpt6
P167
R294
Y173
P304
Rpt4

Supplementary Figure 3. The cryo-EM densities of distinct conformational states.
(**a**) The density map of the S_A state is shown in a transparent gray isosurface and superimposed with the corresponding atomic model. The left shows the complete perspective, whereas the right shows the central slice.
(**b**) Representative secondary structural elements in the S_A state are superimposed with the corresponding cryo-EM densities shown in blue meshes.
(**c**) The density map of the S_B state is shown in a transparent gray isosurface and superimposed with the corresponding atomic models. The left shows the complete perspective, whereas the right shows the central slice.
(**d**) The density map of the S_C state is shown in a transparent gray isosurface and superimposed with the corresponding atomic models. The left shows the complete perspective, whereas the right shows the central slice.
(**e-g**) The density maps of the S_{D1} (panel **e**), S_{D2} (panel **f**) and S_{D3} (panel **g**) state is shown in transparent gray isosurfaces and superimposed with the corresponding atomic models. Cryo-EM densities of representative secondary structural elements are shown in the lower panel.

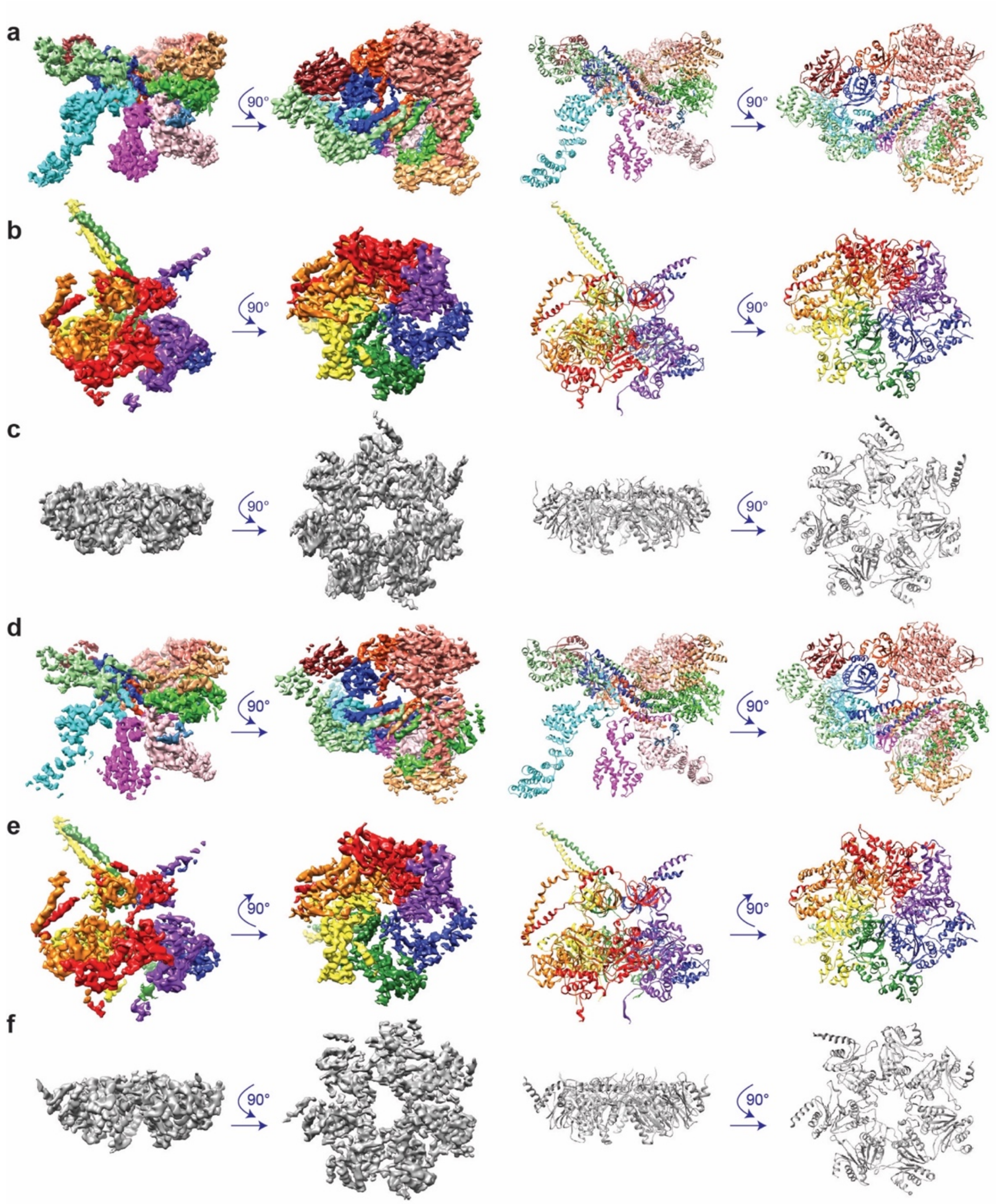

Supplementary Figure 4. The cryo-EM densities of subcomplexes in the S_{D1} and S_{D2} states.
(**a**) Cryo-EM density map of the lid in S_{D1}. The atomic model is shown on the right. In each case, two perpendicular views are shown.
(**b**) Cryo-EM density map of the AAA-ATPase in S_{D1}. The atomic model is shown on the right. In each case, two perpendicular views are shown.
(**c**) Cryo-EM density map of the α-ring in S_{D1}. The atomic model is shown on the right. In each case, two perpendicular views are shown.
(**d**) Cryo-EM density map of the lid in S_{D2}. The atomic model is shown on the right. In each case, two perpendicular views are shown.
(**e**) Cryo-EM density map of the AAA-ATPase in S_{D2}. The atomic model is shown on the right. In each case, two perpendicular views are shown.
(**f**) Cryo-EM density map of the α-ring in S_{D2}. The atomic model is shown on the right. In each case, two perpendicular views are shown.

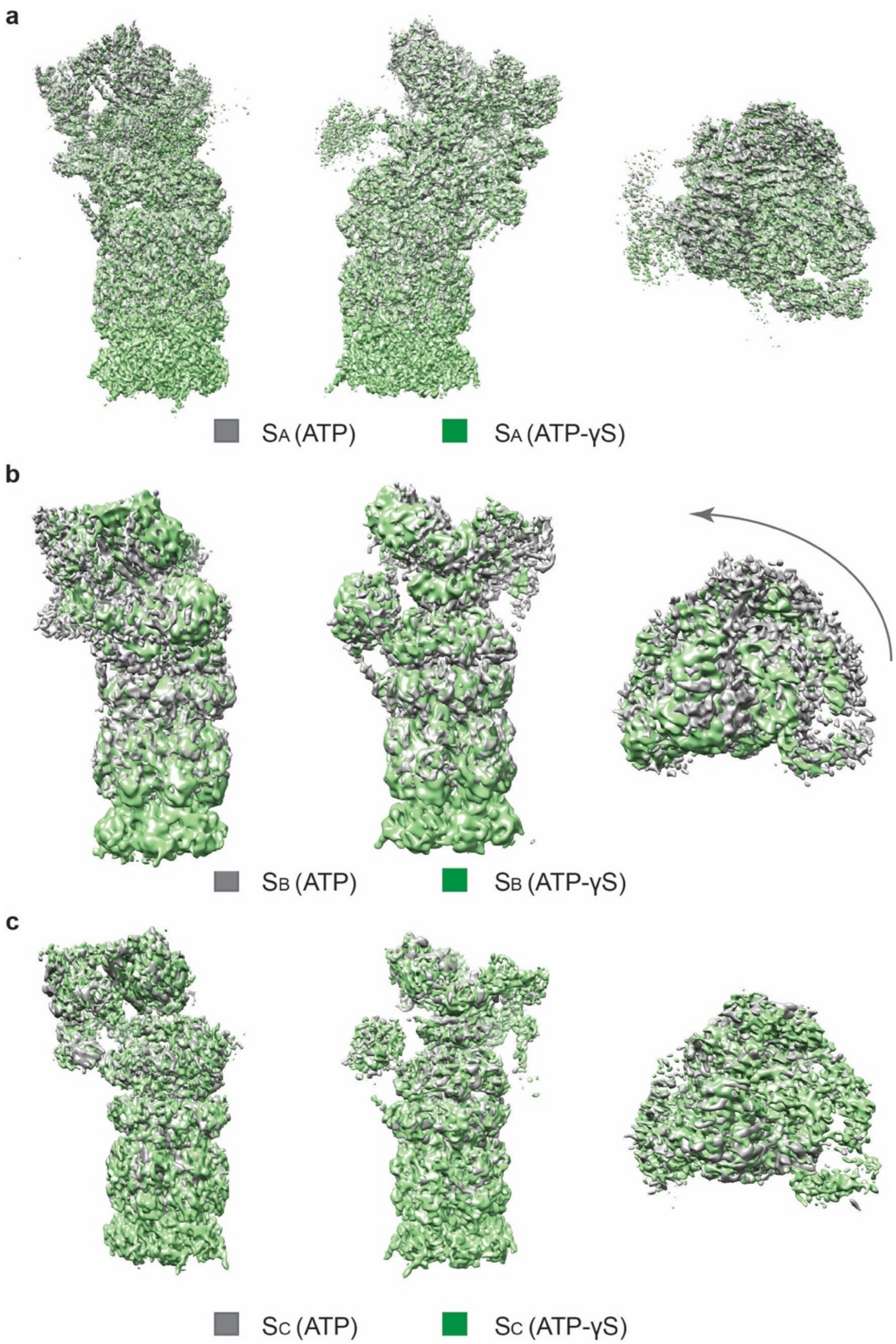

Supplementary Figure 5. Comparison of the ATP-γ-S-bound human 26S structures in the $S_{A,B,C}$ states with those from the ATP-bound ones and among themselves.
(**a-c**) Cryo-EM maps of the present ATP-γ-S-bound human 26S (green) and ATP-bound ones (gray) in S_A (panel **a**), S_B (panel **b**), S_C (panel **c**) states are overlaid and shown in two perpendicular views.

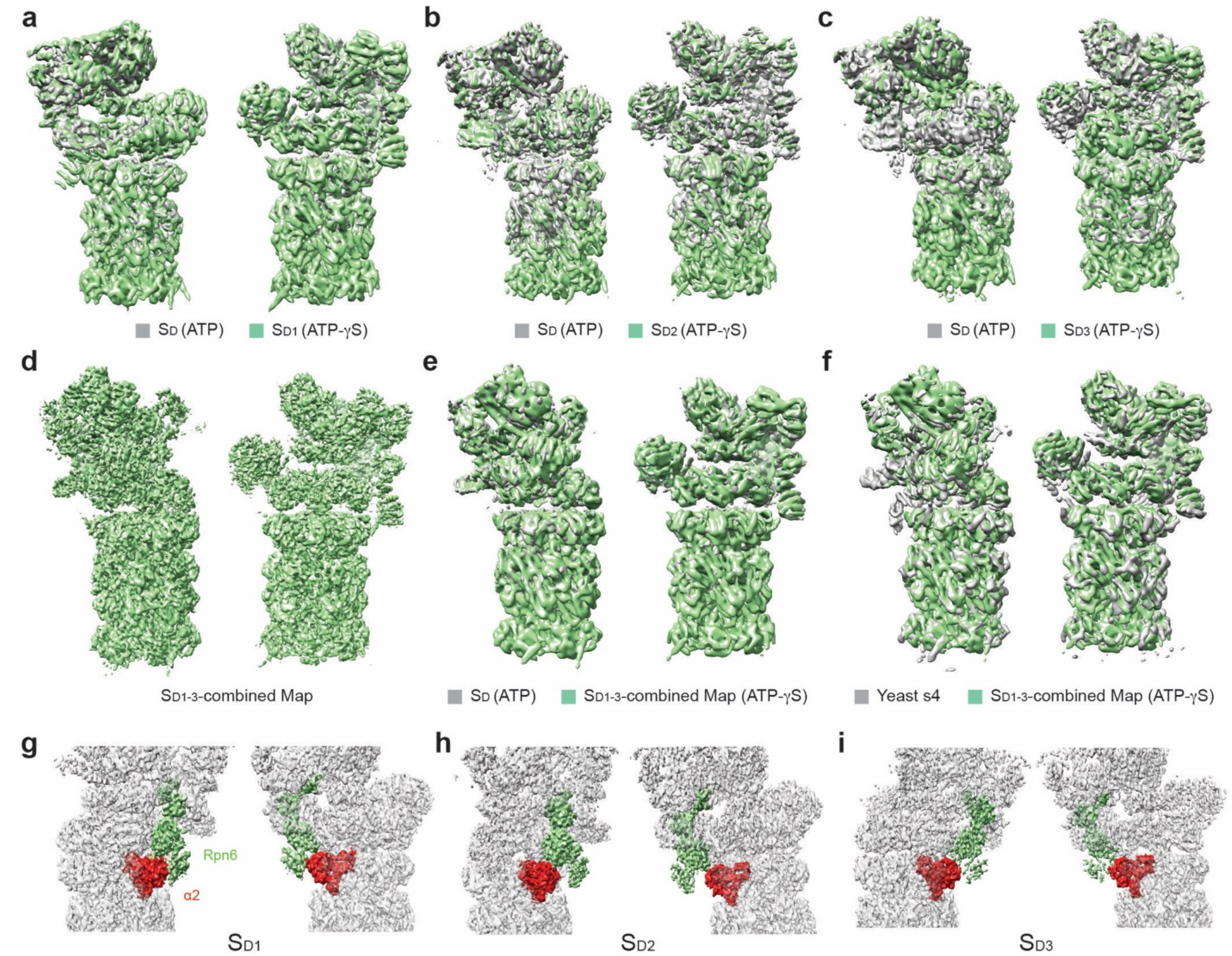

Supplementary Figure 6. Comparison of the ATP-γ-S-bound human 26S structures in different states with those from the ATP-bound ones and among themselves.
(**a**-**c**) Cryo-EM maps of the present ATP-γ-S-bound human 26S low-pass filtered to 8 Å (green) in S_{D1} (panel **a**), S_{D2} (panel **b**), S_{D3} (panel **c**) states are compared with the ATP-bound S_D state.
(**d**) The combined $S_{D1\text{-}3}$ map of the ATP-γ-S-bound human 26S in two perpendicular views.
(**e**) The combined $S_{D1\text{-}3}$ map of the ATP-γ-S-bound human 26S low-pass filtered to 8 Å is superimposed with the ATP-bound S_D map.
(**f**) The combined $S_{D1\text{-}3}$ map of the ATP-γ-S-bound human 26S low-pass filtered to 8 Å is superimposed with the yeast s4 map.
(**g**-**i**) The relative positions of the Rpn6 interacting with the CP subunit α2 in three S_D states. The cryo-EM density maps of the Rpn6 interacting with the CP subunit α-2 of the present ATP-γ-S-bound human 26S in S_{D1} (panel **g**), S_{D2} (panel **h**) and S_{D3} (panel **i**) states respectively.

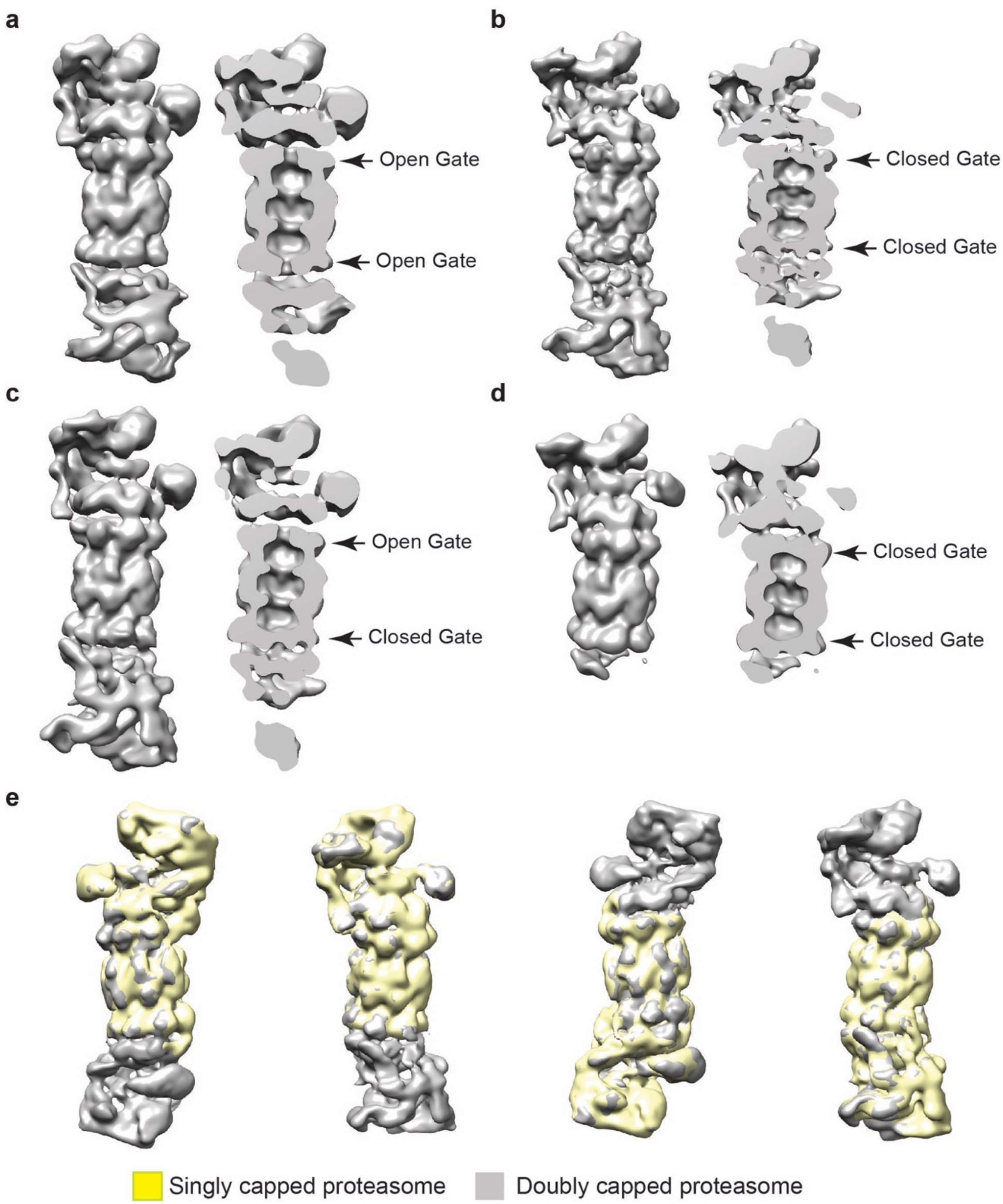

Supplementary Figure 7. **Comparison of the ATP-γ-S-bound human 26S reconstructions in singly and doubly capped forms resulting from 3D classification.**

(a-c) The doubly capped proteasome reconstructions, resulting from 3D classification, with the CP gates both open (panel **a**), both closed (panel **b**), or only one open (panel **c**).

(**d**) The singly capped proteasome reconstruction resulting from 3D classification showed that both CP are closed.

(**e**) The superposition of the singly capped proteasome reconstruction with the doubly capped proteasome with both CP gate closed suggests that they are in the same conformation.

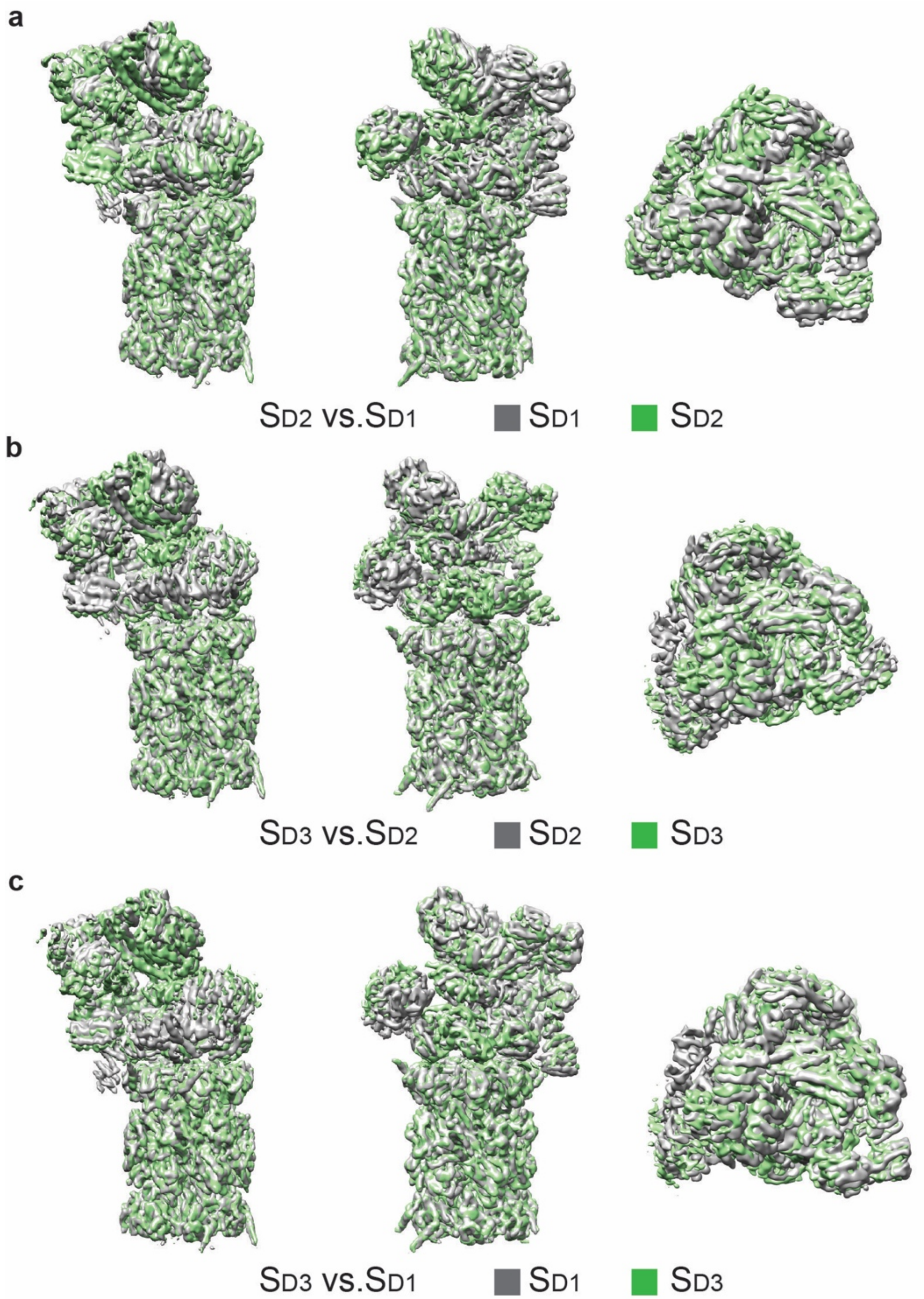

Supplementary Figure 8. Comparison of the cryo-EM maps corresponding to the three S_D states.
The unsharpened maps of S_{D1}, S_{D2} and S_{D3} are superimposed with those of S_{D2} (panel **a**), S_{D3} (panel **b**) and S_{D1} (panel **c**), respectively.

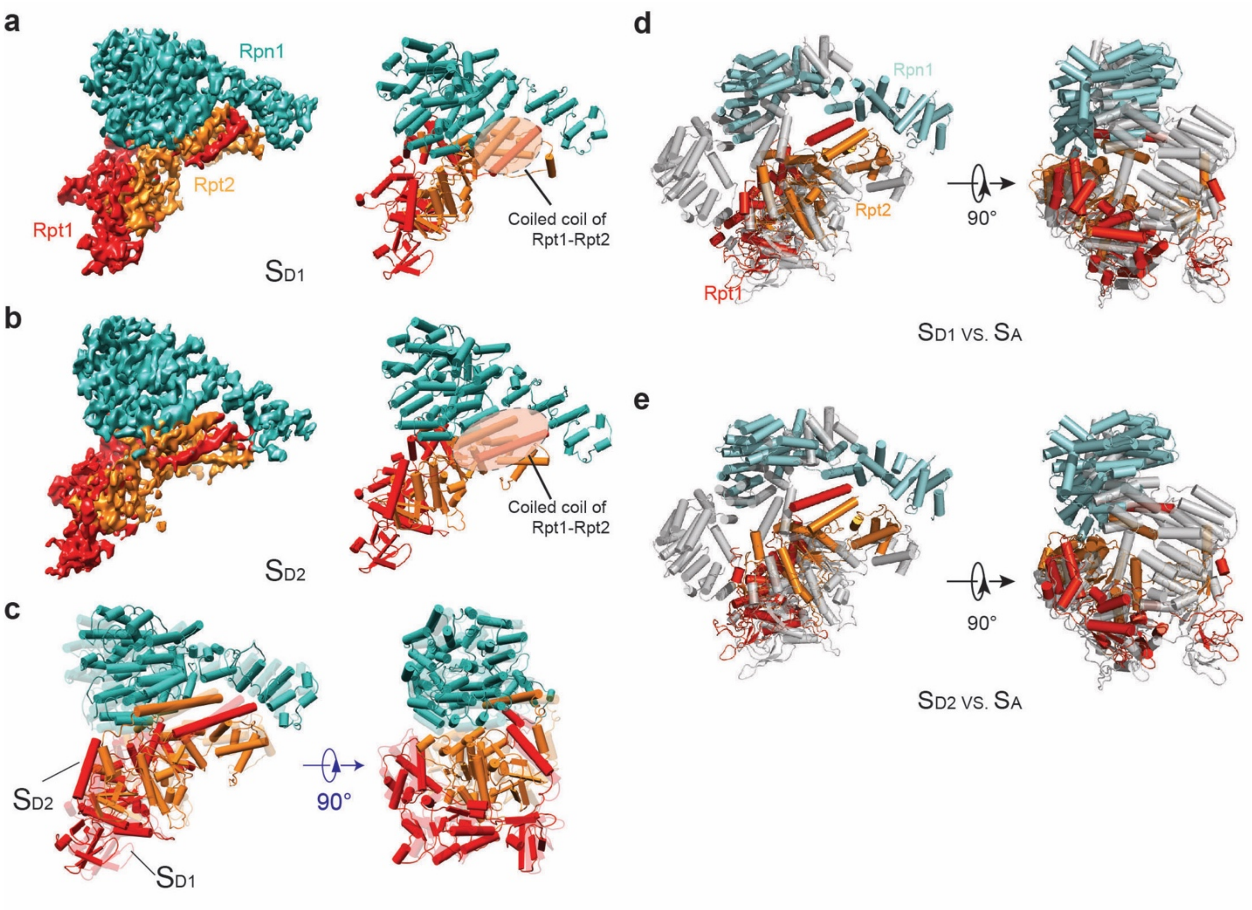

Supplementary Figure 9. Comparison of the Rpn1 models in different states.
(**a**, **b**) The density maps (left) and pseudo-atomic models (right) of Rpn1 (cyan) interacting with Rpt1 (red) and Rpt2 (orange) in the ATPase ring in the S_{D1} and S_{D2} states, respectively, showing the association between Rpn1 and the ATPase ring through coiled coils from Rpt1 and Rpt2. (**c**) Superimposition of the Rpn1-Rpt2-Rpt1 models in the S_{D1} and S_{D2} states in two different views. (**d**) The pseudo-atomic model of Rpn1 in S_{D1} (colored) in superimposed with that in S_A (grey). (**e**) The pseudo-atomic model of Rpn1 in S_{D2} (colored) in superimposed with that in S_A (grey). All models were aligned based on their positions relative to the CP structure.

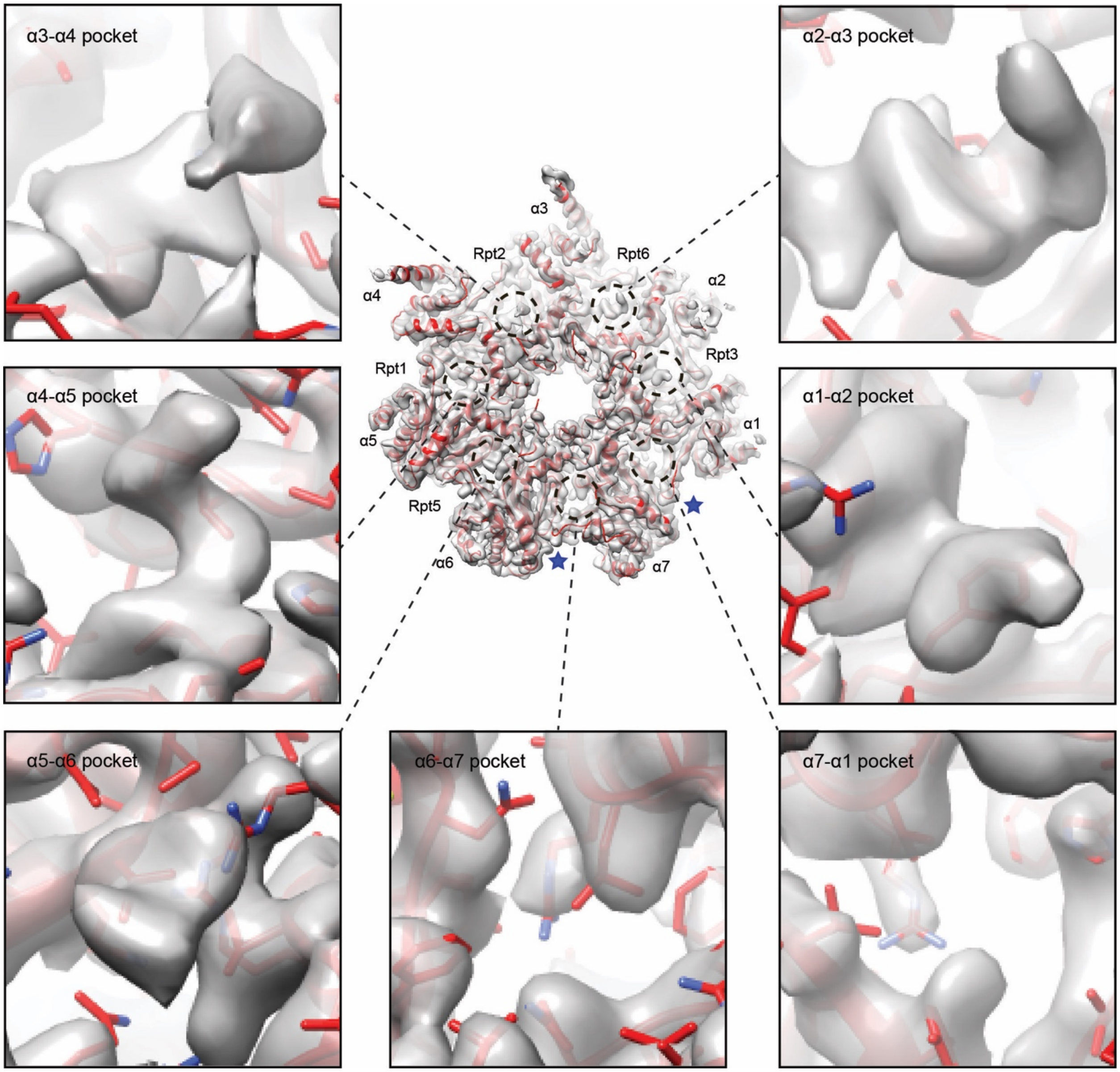

Supplementary Figure 10. The comparison of the extra densities in the α-pockets of the CP that belong to the C-terminal tails of Rpt subunits.

There are seven α-pockets formed on the surface of the CP facing the AAA-ATPase, marked by the dashed circles. Two empty α-pockets are labeled with blue asterisks, what are the α1-α2 and α7-α1 pockets. The cryo-EM density of the CP is rendered transparent and superimposed with the ribbon representation of the atomic model of the CP. The atomic models of Rpt C-terminal tails are not shown to allow a better visual comparison.

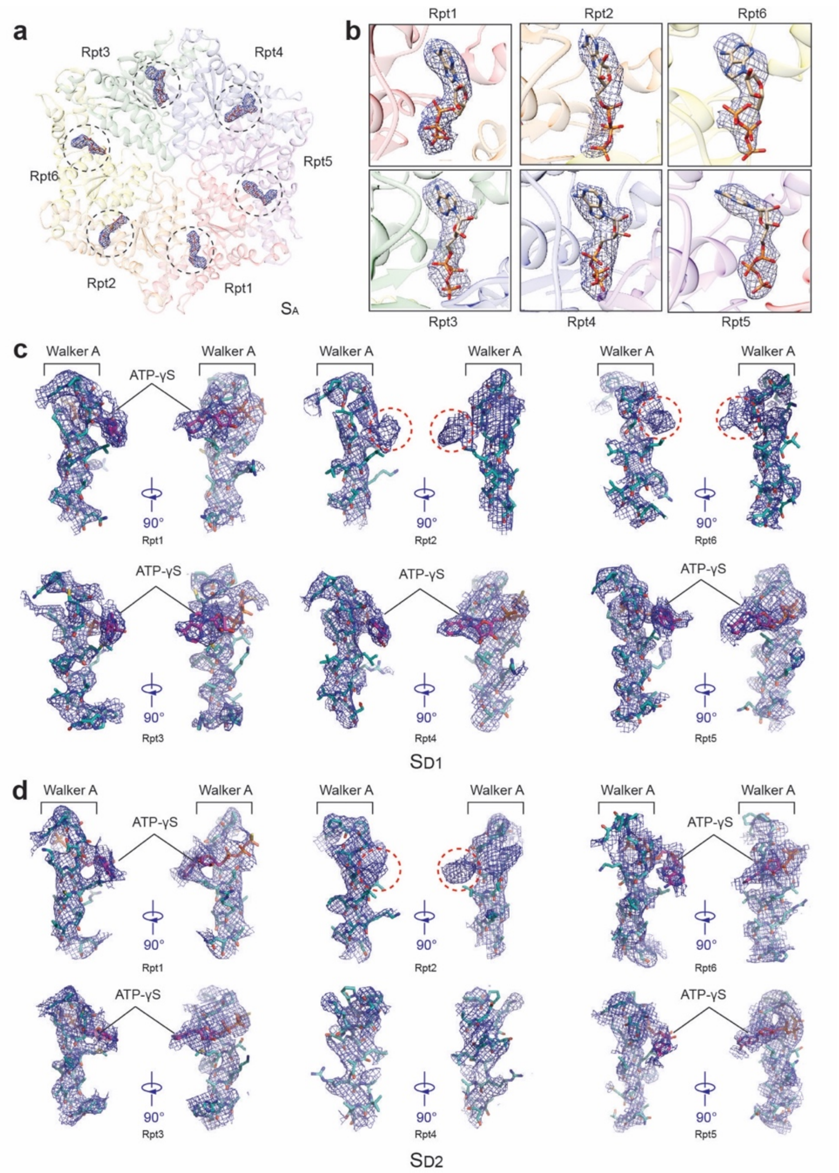

Supplementary Figure 11. The cryo-EM densities of the nucleotides in the ATP-γ-S-bound human 26S proteasome in different states.

(**a**) Overview of six nucleotide-binding sites in the AAA-ATPase heterohexamer of the ATP-γ-S-bound human 26S proteasome in the S_A state. The atomic models of bound nucleotides are shown in a stick representation superimposed with cryo-EM densities of the nucleotides in blue meshes.

(**b**) Close-up views of nucleotide conformations in the six nucleotide-binding sites in the S_A state. ATP-γ-S is modeled into the nucleotide density of each Rpt subunit in the S_A state.

(**c** and **d**) Nucleotides-binding sites in nucleotide-binding pocket of individual AAA-ATPase subunit in the S_{D1} (panel **c**) and S_{D2} (panel **d**). For each Rpt subunit, the cryo-EM density, shown as blue mesh, is superimposed with the atomic model of the nucleotide, the Walker A motif and the α-helix directly extended from the Walker A motif. The densities retained at the same level (8σ) in all sub-panels. The red dashed circles label the in-sufficient densities next to the Walker A motif, in which case the atomic model of ATP-γ-S cannot be fitted into the density. The Walker A motif, shown as a hairpin-turn structure, was labeled in the first row of each panel.

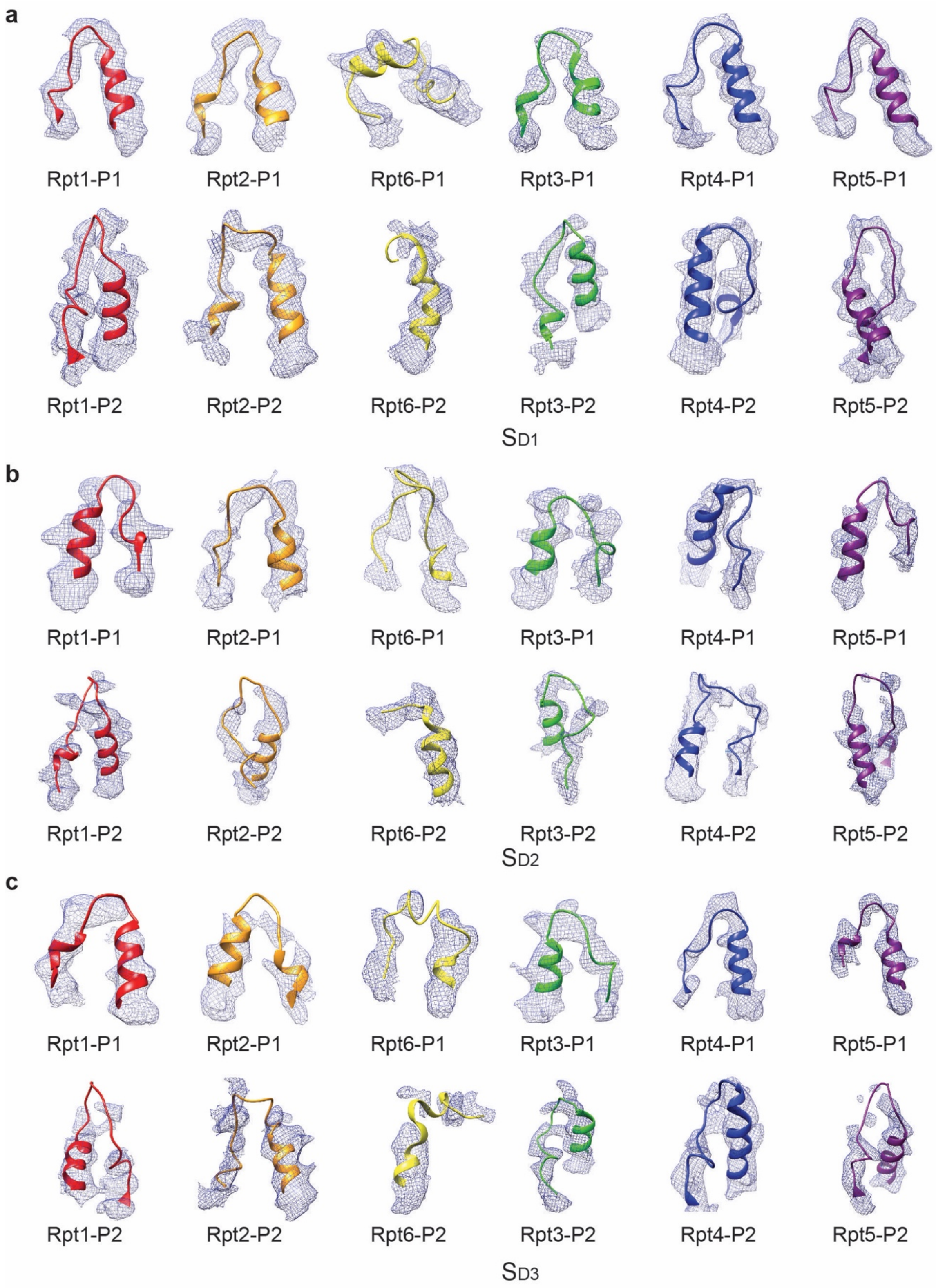

Supplementary Figure 12. The cryo-EM densities of the pore loops.
The cryo-EM densities of pore-1 and pore-2 loops, shown in mesh representation, are superimposed with their atomic models in cartoon representation in the upper and lower rows in each panel, respectively, for the S_{D1} (panel **a**), S_{D2} (panel **b**) and S_{D3} (panel **c**) states.

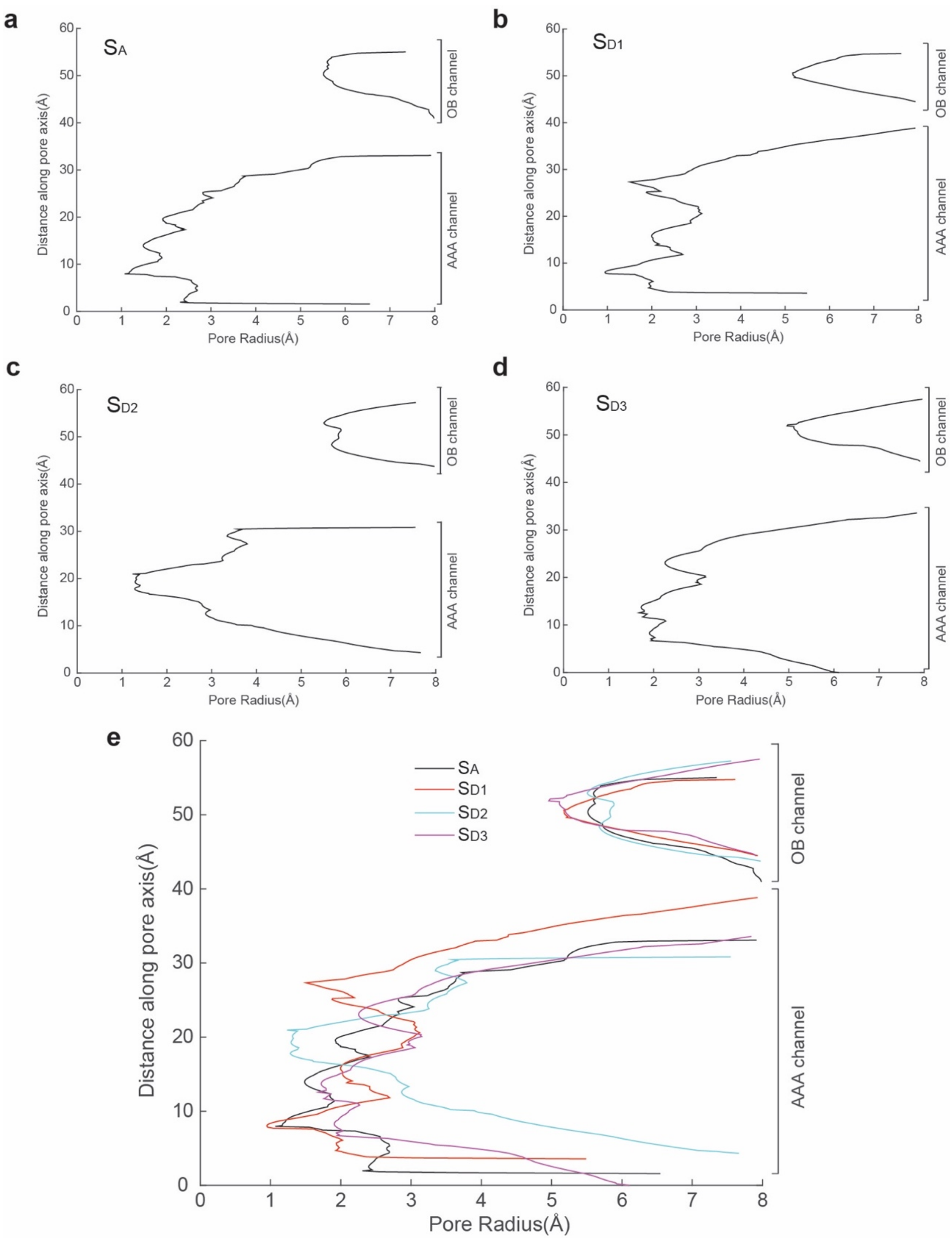

Supplementary Figure 13. The channel radius along the pore axis in the S_A, S_{D1}, S_{D2} and S_{D3} states.

(**a-d**) The channel radius along the pore axis approximately estimated by HOLE in the S_A, S_{D1}, S_{D2} and S_{D3} states.

(**e**) Four curves plotted in one panel shows up and down motions for each state in the AAA-ATPase part.

Supplementary Table 1. Statistics of the ATP-γ-S-bound human 26S proteasome structure determination by single-particle cryo-EM.

**Statistics of the ATP-γ-S-bound human 26S proteasome structure determination by single-particle cryo-EM**							
Electron energy(kV)	200						
Electron dose (e-/ Å2)	30						
Pixel size corresponding to the physical detector sensor (Å)	1.50						
Pixel size in the super-resolution counting mode of K2 Summit (Å)	0.75						
Defocus range (μm)	-3.0 ~ -0.7						
Number of micrographs	8463						
Number of particles for ATPγS-bound 26S proteasome	502,384						
Pseudo Single-Particle	S_A state	S_B state	S_C state	S_{D1} state	S_{D2} state	S_{D3} state	S_D CP
Particle number	214,251	15,536	23,567	66,246	75,726	33,278	175,250
Resolution (Å)	3.6	7	5.8	4.2	4.3	4.9	3.5
B-factor (Å2)	70	50	50	70	80	80	80
Pseudo-crystallographic refinement of atomic models							
Cell dimension a,b,c (Å)	420,420,420			420,420,420	420,420,420	420,420,420	420,420,420
Cell angle α, β, γ (Å)	90,90,90			90,90,90	90,90,90	90,90,90	90,90,90
Space group	P1			P1	P1	P1	P1
Resolution range	420-3.6			420-4.2	420-4.3	420-4.8	420-3.5
Number of atoms	101,280			101,903	100,592	100,821	47,328
R(work) factor				0.3608	0.3265	0.3146	0.334
Geometric Parameters (RMSD)							
Bond length (Å)	0.01			0.01	0.01	0.01	0.01
Bond angles (°)	1.17			1.16	1.19	1.2	0.97
Ramachandran plot statistics							
Favored (%)	92.85			89.13	88.83	87.89	92.53
Allowed (%)	6.77			10.48	11.01	11.65	7.37
Outliers (%)	0.38			0.39	0.16	0.46	0.1
MolProbity validation							
Rotamer outliers (%)	0.49			0.46	0.64	0.59	0.51
Clash score	8.81			10.46	12.58	14.55	5.42
Estimated accuracy in Auto-Refinement in the last iteration							
Accuracy angles (°)	0.993			1.092	1.348	1.468	1.439
Accuracy offsets (Pixel)	0.605			0.715	0.8	0.93	0.784
