# Supplementary material for: Structural mechanism for nucleotide-driven remodeling of the AAA-ATPase unfoldase in the activated human 26S proteasome
Source: Nat Commun. 2018 Apr 10;9:1360. doi: 10.1038/s41467-018-03785-w (PMC5893597; doi:10.1038/s41467-018-03785-w)
Supplement: Supplementary file 3 — Description of Additional Supplementary Info [file 41467_2018_3785_MOESM3_ESM.pdf]

Description of Additional Supplementary Files

File Name: Supplementary Movie 1
Description: Overall conformational change of the open-gate states. A movie clip that shows the conformational change of the AAA-ATPase in the ATP-γS-bound human proteasome.

File Name: Supplementary Movie 2
Description: Motion of the pore-1 loops of the open-gate states. A movie clip that shows the structural changes of the pore-1 loops in the AAAATPase unfoldase.

File Name: Supplementary Movie 3
Description: Motion of the pore-2 loops of the open-gate states. A movie clip that shows the structural changes of the pore-2 loops in the AAAATPase unfoldase.
