# Supplementary material for: Structural mechanism for nucleotide-driven remodeling of the AAA-ATPase unfoldase in the activated human 26S proteasome
Source: Nat Commun. 2018 Apr 10;9:1360. doi: 10.1038/s41467-018-03785-w (PMC5893597; doi:10.1038/s41467-018-03785-w)
Supplement: Supplementary file 2 — Peer Review Report [file 41467_2018_3785_MOESM2_ESM.pdf]

This manuscript has been previously reviewed at another journal that is not operating a transparent peer review scheme. This document only contains reviewer comments and rebuttal letters for versions considered at Nature Communications. Mentions of prior referee reports have been redacted.

Reviewers' comments:

Reviewer #1 (Remarks to the Author):

The authors revised the manuscript according to the comments raised earlier. The manuscript was improved and the discussions are somewhat more objective. However, the concerns persist regarding an over-interpretation of the data. The authors described different conformations that appear to be associated with an open gate state of the proteasome. However, the data does not seem sufficiently strong for an unambiguous interpretation of the nucleotide densities to justify a direct association between nucleotide occupancy and those conformational states. With the existing data, a clear focus on the conformational variants observed, with a stripping down of the speculations on nucleotide occupancy, would result in a stronger manuscript albeit of a not so strong impact.

The manuscript describes RP conformations that the authors attribute to variability of nucleotide occupancy. In the previous version of the manuscript the authors showed in a figure the nucleotide binding sites, but the densities corresponding to the protein densities, namely alpha helices, did not appear compatible with the resolution required to resolve such details. The authors now provide a new Supplementary Figure 11 were the maps shown have been sharpened. At a resolution of 4-5 Angstroms, as claimed, the protein backbone should be unambiguously traced and densities for side chains, particularly bulky ones, should be observed. However, the new figures panels e-g appear to show a mismatch between the map densities and the protein models, which are shown as cartoons with no side chains represented. The protein densities do not follow the helical pattern of the model and it is speculative if the densities identified correspond to nucleotide densities, as it not clear that these correspond to real map densities not accounted for by the protein model. It is also quite intriguing why the nucleotide appears to take quite distinct conformations at the Rpt1, Rpt2, Rpt3 and Rpt5 sites in the SA state (Supplementary Figure 11b) compared to the respective sites in the SD3 state (Supplementary Figure 11d). This needs to be clarified and carefully justified. Overall the densities for Rpn1 in the different maps also appear too weak for accurate modelling and to justify their interpretation over previously published data.

The merging of particle sub-sets, or class averages, should be done on an objective, quantitative, basis not just on a subjective overall judgement of visual appearances. Therefore the argument of

including an additional class in the classification of the SD states based on an apparent open gate is not strong. This also applies to the merging of single capped complexes in the analysis of the SA state. Curiously, in the new Supplementary Figure 7d the authors show their 3D map of a single capped proteasome complex. However, densities clearly extend from the CP at the opposite end to the RP. Are these due to incomplete classification of RP occupancy or do they correspond to different complexes? Can these therefore be properly merged with the SA state?

Point-by-Point Response to Reviewers' Comments:

Reviewer #1:

Comments: The authors revised the manuscript according to the comments raised earlier. The manuscript was improved and the discussions are somewhat more objective. However, the concerns persist regarding an over-interpretation of the data. The authors described different conformations that appear to be associated with an open gate state of the proteasome. However, the data does not seem sufficiently strong for an unambiguous interpretation of the nucleotide densities to justify a direct association between nucleotide occupancy and those conformational states. With the existing data, a clear focus on the conformational variants observed, with a stripping down of the speculations on nucleotide occupancy, would result in a stronger manuscript albeit of a not so strong impact.

Response: We thank the reviewer for pointing this out. In the revised manuscript, we have generally taken the reviewer's suggestion in several ways:

(1) We completely removed any discussion or speculation regarding the nucleotide occupancy of the S_{D3} state from both main text and Supplementary Information; We avoided making any speculation nor discussion regarding the nucleotide occupancy in the S_{D3} state.

(2) To improve the identification of the nucleotide states in the S_{D1} and S_{D2} conformations, we improved the local density quality of ATPase subunits in the S_{D1} and S_{D2} states by furthering refinement using a local mask centered at the AAA-ATPase module and CP, which improve the gold-standard resolution of ATPase/CP in the S_{D1} and S_{D2} states to 4.0 and 4.1 Å, respectively. To better illustrate this, we revised Supplementary Figure 11c, d accordingly as well as the Methods section (see page 16).

(3) For the S_{D1} and S_{D2} states, we have downgraded our conclusion to that "at least four nucleotides are bound to the AAA-ATPase ring in these open-gate states", and explicitly pointed out "either a partial occupancy or disordered nucleotide configuration" within the remaining two nucleotide-binding sites in the S_{D1} and S_{D2} states.

Comments: The manuscript describes RP conformations that the authors attribute to variability of nucleotide occupancy. In the previous version of the manuscript the authors showed in a figure the nucleotide binding sites, but the densities corresponding to the protein densities, namely alpha helices, did not appear compatible with the resolution required to resolve such details. The authors now provide a new Supplementary Figure 11 were the maps shown have been sharpened. At a resolution of 4-5 Angstroms, as claimed, the protein backbone should be unambiguously traced and densities for side chains, particularly bulky ones, should be observed. However, the new figures panels e-g appear to show a mismatch between the map densities and the protein models, which are shown as cartoons with no side chains represented. The protein densities do not follow the helical pattern of the model and it is speculative if the densities identified correspond to nucleotide densities, as it not clear that these correspond to real map densities not accounted for by the protein model. It is also quite intriguing why the nucleotide appears to take quite distinct conformations at the Rpt1, Rpt2, Rpt3 and Rpt5 sites in the SA state (Supplementary Figure 11b) compared to the respective sites in the SD3 state (Supplementary Figure 11d).

Response: In the revised manuscript, we have improved the local density quality of ATPase subunits in the S_{D1} and S_{D2} states by furthering refinement using a local mask centered at the AAA-ATPase module and CP, which made it clearer that at least four nucleotide-binding sites are occupied in the S_{D1} and S_{D2} states. We revised Supplementary Figure 11 accordingly. The nucleotide binds the Walker A motif in the ATPase subunit, which is a hairpin fold following a conserved pattern of amino acid sequence "Gly-Pro-Pro-Gly-Thr-Gly" that all have small side chains. Following the Walker A motif is a conserved alpha helix (Lys-Thr-Leu/Met-Leu-Ala) that contains small side chains in majority. The lysine residue is the only large

side chain here potentially being confused with nucleotide density during map interpretation; however, the nucleotide density is about three to four-time larger than that of a lysine side chain. For example, in the revised Supplementary Fig. 11c, the helix next to the Walker A motif in Rpt1/3/4/5 are clearly resolved with prominent helical groove, making it very intuitive to see the striking density of nucleotides. The local density quality in Rpt2 and Rpt6 around the Walker A motif is clearly worse than the others (Supplementary Fig. 11c). To avoid over-interpretation, we did not make definite conclusions about the nucleotide states in the remaining two ATPase subunits, but instead stating multiple possibilities, that is, "either a partial occupancy or disordered nucleotide configuration within these nucleotide-binding sites". See page 9. Lastly, to practice caution and rigor, we have completely removed the description of nucleotide-binding state of S_{D3} in the main text, as well as removed the previous Supplementary Fig. 11d and g.

Comments: Overall the densities for Rpn1 in the different maps also appear too weak for accurate modelling and to justify their interpretation over previously published data.
Response: We have taken this suggestion into account and downgraded the claim of Rpn1 modeling in the revision. We downgraded the section "Rpn1 engages the AAA-ATPase unfoldase via coiled-coil domains" into a short paragraph on page 7. The original Figure 4 is moved from the main text to the newly revised Supplementary Fig. 9.

Comments: The merging of particle sub-sets, or class averages, should be done on an objective, quantitative, basis not just on a subjective overall judgement of visual appearances. Therefore the argument of including an additional class in the classification of the SD states based on an apparent open gate is not strong. This also applies to the merging of single capped complexes in the analysis of the SA state.
Response: To address this concern, we have done a reconstruction of the merged S_D state by excluding the additional 4th class, which reduces the number of particles by only about 20%. The resulting reconstruction of the CP is still measured to be 3.5 Å by gold-standard FSC and is correlated at a coefficient of 0.993 with the previous CP reconstruction that included the 4th class (see fig. a, b below). We have accordingly revised the Methods section regarding the merged S_D state without including the 4th class. All conclusions related to the CP reconstruction of the S_D state remain unchanged.

Comments: Curiously, in the new Supplementary Figure 7d the authors show their 3D map of a single capped proteasome complex. However, densities clearly extend from the CP at the opposite end to the RP. Are these due to incomplete classification of RP occupancy or do they correspond to different complexes? Can these therefore be properly merged with the SA state?
Response: We have completed a reconstruction of the S_A state by excluding the singly-capped proteasome, which reduces the particle number from 241329 to 214251. The resulting map is still measured to 3.6 Å by the gold-standard FSC; it correlates to 0.997 with the previous S_A map and exhibits the same high-resolution features (see fig. c, d below). We accordingly updated the Methods section, Supplementary Figure 2 and Table 1. Note that merging the singly capped complex with the S_A state was previously practiced by others and have shown the effect of improving resolution (see Ref. 29, Schweitzer et al. PNAS, 113, 7816, 2016).

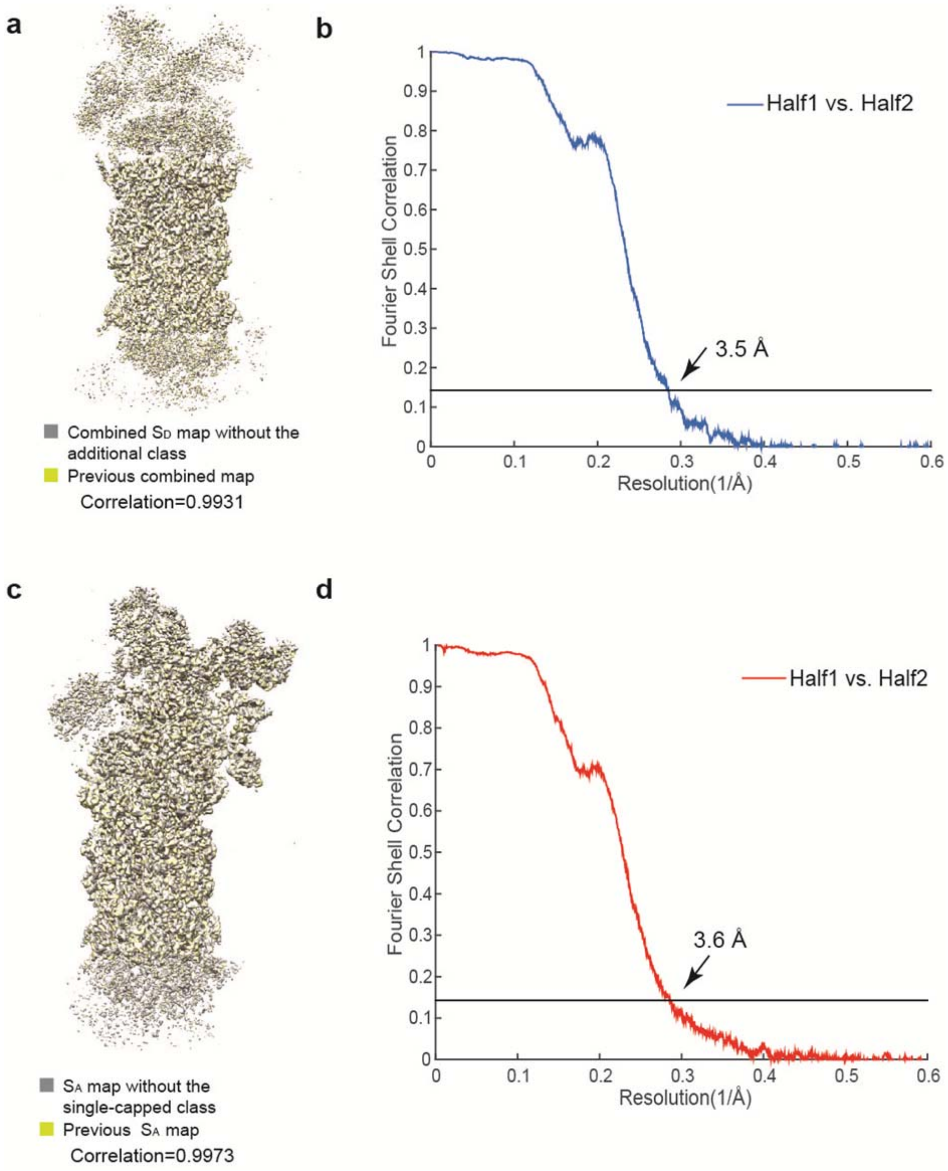

a
Combined SD map without the additional class
Previous combined map
Correlation=0.9931
b
Half1 vs. Half2
Fourier Shell Correlation
3.5 Å
Resolution(1/Å)
0
0.1
0.2
0.3
0.4
0.5
0.6
0.7
0.8
0.9
1
c
SA map without the single-capped class
Previous SA map
Correlation=0.9973
d
Half1 vs. Half2
Fourier Shell Correlation
3.6 Å
Resolution(1/Å)

REVIEWERS' COMMENTS:

Reviewer #1 (Remarks to the Author):

The authors adequately addressed the issues raised and, by down-weighting their previous highly speculative interpretations of the results, successfully improved the manuscript.
